# DYNAMIC: A Novel Software Implementation of a Kinetic Model of TaqMan PCR

**DOI:** 10.1021/acs.analchem.5c07375

**Published:** 2026-03-16

**Authors:** Louis Kreitmann, Ye Mao, Ke Xu, Alison Holmes, Karen Brengel-Pesce, Laurent Drazek, Jesus Rodriguez-Manzano

**Affiliations:** † Department of Infectious Disease, 4615 Imperial College London, London W12 0NN, U.K.; ‡ 1896 Open Innovation & Partnerships, bioMérieux, 376 Chemin de l’Orme, Marcy-l'Étoile 69280, France; § Department of Electrical and Electronic Engineering, 4615 Imperial College London, London W12 0NN, U.K.; ∥ The Fleming Initiative, Imperial College London and Imperial College Healthcare NHS Trust, London W2 1NY, U.K.; ⊥ Molecular Biology, Research & Development, bioMérieux, 5 Rue des Berges, Grenoble 38000, France; # Data Science, Research & Development, bioMérieux, 5 Rue des Berges, Grenoble 38000, France

## Abstract

Multiplex PCR is a key modality of nucleic acid amplification testing with growing applications in clinical diagnostics, especially in infectious diseases. Recent work has demonstrated that thermodynamic and kinetic information embedded in amplification curves (ACs) can be leveraged for target identification in the multiplex setting. This technology, named Amplification Curve Analysis (ACA), requires a mechanistic simulation tool linking biochemical design choices to AC features. We present DYNAMIC, an open-source Python implementation of a kinetic model acting as a digital twin of singleplex TaqMan PCR. Based on established kinetic and stoichiometric principles, DYNAMIC predicts fluorescence values over a wide range of experimental conditions. Key features include separate modeling of primer and probe annealing, a flexible 2-parameter thermal degradation model of Taq activity, and support for atypical regimes relevant to ACA, such as asymmetric primer concentrations. A global optimization algorithm identifies thermodynamic hyperparameters linking assay characteristics to AC features. In comparison with experimental data, DYNAMIC reproduces AC variations driven by changes in primer and probe concentrations, captures late-cycle efficiency loss from enzyme degradation, and yields realistic cycle threshold trends across orders of magnitude in input DNA. Tested against a dilution series of four previously published assays, the model robustly identifies key kinetic hyperparameters. Overall, DYNAMIC provides a mechanistic framework for predicting TaqMan PCR kinetics that can streamline assay development, reduce empirical optimization, and support the rational design of multiplex panels where target identification relies on AI-enabled classification of AC features.

## Graphical abstract

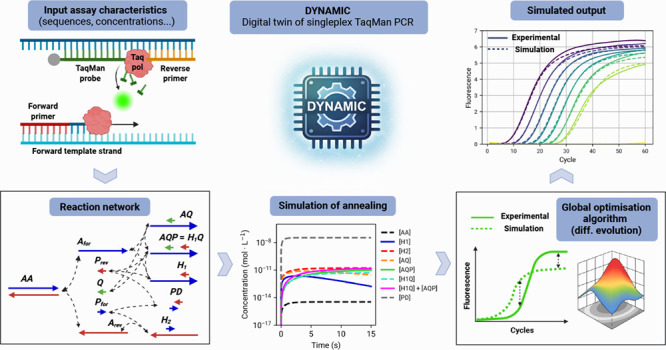

## Introduction

Multiplex polymerase chain reaction (mPCR) enables the simultaneous detection and quantification of multiple DNA or RNA targets in a single reaction and has broad applications in medical diagnostics, environmental surveillance, and molecular biology research. Several strategies have been developed to enhance the multiplexing capabilities of PCR-based assays. These can be broadly categorized into spatial multiplexing and single-well technologies.^1^ Spatial multiplexing techniques leverage microfluidics for the spatial segregation of PCR, enabling parallel yet spatially distinct amplification and detection of different targets. Commercial platforms based on these techniques are particularly useful at the point-of-care, but may have intrinsic limitations—including their relatively high cost, low throughput, and the requirement for specialist equipment—which can limit their deployment in some clinical settings.^2,3^

Conversely, single-well techniques have in common that all primer pairs are combined in the same PCR mixture at the onset of the reaction.^4^ Here, the main challenge (besides assay design) lies in the correct identification of amplified products. Two widely used solutions are melting curve analysis (MCA) and probe-based assays (the most common method being TaqMan hydrolysis probes). MCA relies on the melting behavior of double-stranded DNA: progressive heating of end-amplification products leads to a characteristic loss-of-fluorescence curve (the melting curve; MC) which can be mapped specifically to amplicon sequence.^5,6^ In contrast, the TaqMan technology uses sequence-specific fluorescent probes that hybridize to target regions during amplification. Cleavage of the probe by the DNA polymerase releases a reporter signal, enabling real-time, highly specific detection. By combining probes labeled with different fluorophores, multiple targets can be detected simultaneously in a single reaction with high sensitivity and specificity.^7^ However, this approach is typically limited to a maximum of five fluorophores (or detection channels) because of potential overlap between emission spectra.^4^

Recently, our group has demonstrated that thermodynamic and kinetic information embedded in amplification curves (ACs)—obtained both with intercalating dyes and TaqMan-based assays—can be leveraged for target identification in the multiplex setting.^8–10^ In amplification curve analysis (ACA), machine learning (ML) models are trained to learn a mapping between ACs and target classes, effectively extracting AC features for accurate classification.^11^ In practice, ACA works best when assays are deliberately engineered to yield maximally separable ACs—a concept termed “tailored chemistry”.^12^ This creates a pressing need for a mechanistic model that links biochemical design choices to AC features, enabling in silico exploration before wet-lab experimentation. Furthermore, such a model could be used to generate a large volume of synthetic yet thermodynamically realistic ACs to train artificial intelligence (AI) models for ACA, which would substantially lower the cost and effort of building experimental training data sets.

Several mathematical models of PCR have been published previously,^13–18^ often to precisely estimate PCR efficiency (and thus improve the quantification of input copy numbers), or predicting the effects of nonspecific DNA interactions. These models aim to solve the system of equilibrium (stoichiometric) and kinetic equations that governs the three fundamental steps of the PCR (i.e., denaturation, annealing, and elongation), enabling the computation of concentrations of all DNA molecules involved (i.e., single- and double-stranded templates, amplicons and oligos) across cycles. While valuable, these frameworks present important limitations: they do not model the annealing process of primers and probes separately; they offer limited control over enzyme activity decay across cycles; and they lack the flexibility required to explore atypical regimes relevant to the design of ACA-based multiplex panels (e.g., asymmetric primer concentrations). Importantly, most existing models focus on the initial amplification steps (usually up to the cycle threshold, or *C*_t_ value), and fail to describe and predict amplification kinetics at later stages of the AC, specifically its linear portion and plateau—both of which are key features in the context of ACA. Moreover, parameter values that govern amplification kinetics are difficult to measure and are rarely calibrated to reproduce full AC shapes across dilution series.

In this work, we describe DYNAMIC, a novel open-source Python implementation of a mathematical model of TaqMan-based PCR. We conduct extensive in silico simulations and apply them to wet-lab experiments to demonstrate their accuracy and versatility. The novelties of our approach include the following: DYNAMIC models the behavior of the forward/reverse primers and probe independently; it incorporates a flexible 2-parameter thermal degradation model of Taq activity across cycles; it supports atypical conditions (e.g., asymmetric PCR, single-stranded DNA templates); it embeds a global optimization algorithm that fits key thermodynamic and kinetic parameters to experimental ACs, yielding predictive, assay-specific simulations over the whole range of PCR cycles; and it allows computing ACs over a range of dilution series (standard curve).

The code is available at https://github.com/lkreitmann-bmx/Dynamic.

## Experimental Section

### DYNAMIC

#### Reaction Network

DYNAMIC is a simulator of singleplex PCR that works by computing the concentrations of 12 molecules across PCR cycles (Figure 1):

- *AA* (double-stranded amplicon)
- *A*_for_ (amplicon forward strand)
- *A*_rev_ (amplicon reverse strand)
- *P*_for_ (forward primer)
- *P*_rev_ (reverse primer)
- *Q* (probe)
- *H*_1_ (*A*_for_ + *P*_rev_ hybrid)
- *H*_2_ (*A*_rev_ + *P*_for_ hybrid)
- *AQ* (*A*_for_ + *Q* hybrid)
- *AQP* (*A*_for_ + *Q* + *P*_rev_ hybrid formed by the annealing of *P*_rev_ to *AQ*)
- *H*_1_ *Q* (*A*_for_ + *Q* + *P*_rev_ hybrid, identical in structure to *AQP*, but formed by the annealing of *Q* to *H*_1_)
- *PD* (primer dimer, i.e., *P*_for_ + *P*_rev_ hybrid).

**Figure 1:**
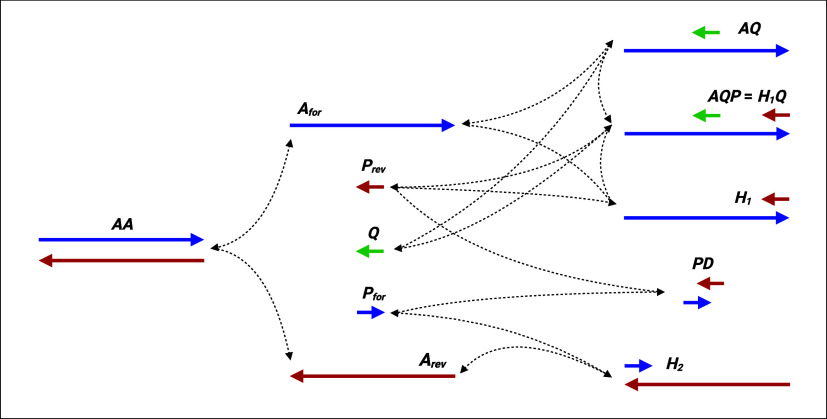
Reaction network of DYNAMIC. This plot shows the 12 molecular species whose concentrations are computed and tracked across PCR cycles by DYNAMIC, as well as their interactions during the annealing step (modeled by the *annealing* function). Note that by convention in DYNAMIC, *Q* anneals to *A*_for_ (hence is in the same direction as *P*_rev_). Single-stranded templates and oligos have arrows indicating the 5′ to 3′ direction. Also, note that the same molecular species *H*_1_ *Q* = *AQP* can be converted to either *AQ* + *P*_rev_ or *H*_1_ + *Q*, and these reactions are modeled separately in the *annealing* function. Finally, dimers involving either *P*_for_ or *P*_rev_ with *Q*, or three-molecular hybrids involving *P*_for_, *P*_rev_, and *Q* are not considered. Figure created with BioRender.

Note that by convention, in DYNAMIC the probe *Q* anneals to *A*_for_ (that is, in the same direction as *P*_rev_; see Figure 1). Sequences for the single-stranded templates, strands, and oligos are input by the user in the 5′ to 3′ direction. To keep the model simple, DYNAMIC does not consider nonspecific (i.e., off-site) interactions of primers on template strands, dimers involving either *P*_for_ or *P*_rev_ with *Q*, or three-molecular hybrids involving *P*_for_, *Q*, and *P*_rev_.

The concentrations of these 12 molecular species are stored in a dictionary called *dict_conc*. Initial values are set by the user and stored in *dict_conc*[0]. The sequences of *A*_for_, *P*_for_, *P*_rev_, and *Q* are stored in a dictionary named *dict_seq*, used to calculate the free energy (Δ*G*) associated with hybridization reactions. Hyperparameters are stored in three dictionaries: *dict_PCR* stores the number of PCR cycles (*N*_cycles_), as well as the duration and temperature of the denaturation, annealing, and elongation steps (*time_denaturation*, *temp_denaturation*, *time_annealing*, *temp_annealing*, *time_extension*, and *temp_extension*); *dict_enzyme* stores the Taq polymerase characteristics, essentially related to its thermal degradation across cycles (detailed below); and *dict_thermo* stores association and dissociation constants (*k*_on_ and *k*_off_) associated with hybridization reactions, including the formation of *H*_1_, *H*_2_, *AQP*, and *H*_1_ *Q* hybrids, the reannealing of *A*_for_ and *A*_rev_ into *AA*, and the formation of *PD*.

#### Running One PCR Simulation

A simple PCR simulation involves running the function *run_PCR*, which itself calls the function *run_1_cycle* *N*_cycles_ times (see Algorithm 1). *run_1_cycle* works by calling iteratively three functions, corresponding to the three steps of a PCR cycle, i.e., *denaturation*, *annealing*, and *elongation* (see Algorithm 2).
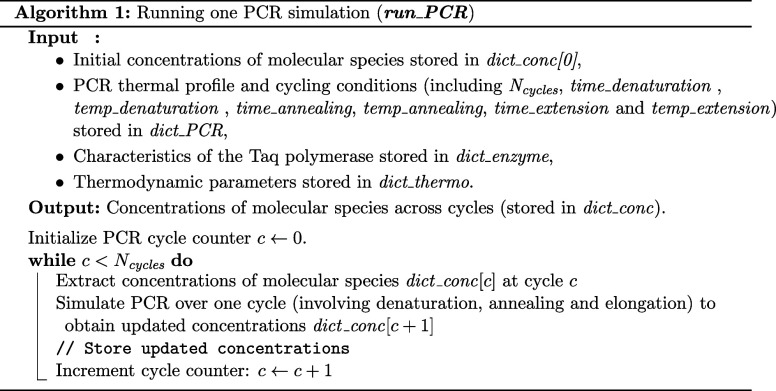
 
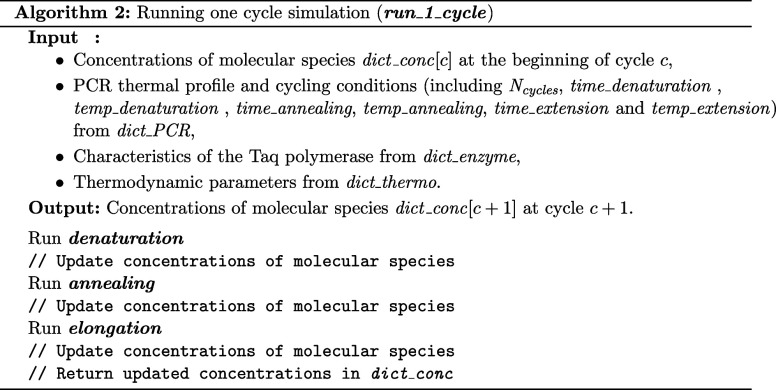


#### Three Key Modeling Functions

*Denaturation* is a simple function where we assume that heating the PCR mix at *temp_denaturation* (usually set at 95 °C) during *time_denaturation* (usually set at 15 s) will result in 100% denaturation of all hybrids into single molecules.^19,20^ For instance, all *AA* is converted to *A*_for_ and *A*_rev_, all *H*_1_ is converted to *A*_for_ and *P*_rev_, etc.

Similarly, *elongation* is a simple function where we assume that the Taq polymerase can fully complete the elongation process from hybrids *H*_1_, *H*_2_, *AQP,* and *H*_1_ *Q* into *AA* at each cycle. Of note, *AQ* is not elongated into *AA* because in TaqMan probes, the 3′-hydroxyl group is deleted to prevent amplification (and enforce degradation of the probe through the nuclease activity of the polymerase). Consistent with the mechanism of fluorescence emission during TaqMan assays,^7^ light emission is recorded when *AQP* and *H*_1_ *Q* (but not *H*_1_ or *H*_2_) are converted into *AA*. DYNAMIC does not include an explicit model for fluorescence emission, one that would capture the nonlinear, instrument- and chemistry-dependent relationship between species concentrations and the measured signal. Instead, it is based on the minimalist assumption that fluorescence increases linearly with probe cleavage, with one arbitrary unit assigned per molecule of probe cleaved by the Taq polymerase through its 5*′* → 3*′* exonuclease activity during elongation.

*Annealing* is the most complex function, as it models the formation of oligo hybrids during the second step of PCR. This function works by solving a system of ordinary differential equations (ODEs) that includes: (1) kinetic equations describing the dynamics of hybrid formation from single-stranded oligos and (2) equilibrium equations enforcing the law of mass conservation within the system.

During the annealing process, the formation of *H*_1_, which results from the binding of *P*_rev_ to *A*_for_ can be represented by the following chemical reaction:

$$A_{\mathrm{for}}+P_{\mathrm{rev}}\overset{k_{1}^{\mathrm{on}}}{\underset{k_{1}^{\mathrm{off}}}{⇌}}H_{1}$$ 1

where

$$K_{1}=\frac{k_{1}^{\mathrm{on}}}{k_{1}^{\mathrm{off}}}$$ 2

and

$$K_{1}=e^{-\Delta G_{1}/RT}$$ 3

with *R* = 8.314 J·mol^–1^·K^–1^ (universal gas constant) and *T* the absolute temperature in Kelvin.

To compute Δ*G*_1_, we use the following formula:

$$\Delta G_{1}=\Delta H_{1}-T\Delta S_{1}$$ 4

where Δ*H*_1_ (variation of enthalpy) and Δ*S*_1_ (variation of entropy) are obtained using the *Tm_NN* function from the *Biopython* package, based on published nearest neighbor thermodynamics databases and formulas.

From this, a first-order kinetic equation can be derived:

$$\frac{d[H_{1}]}{dt}=k_{1}^{\mathrm{on}}[A_{\mathrm{for}}][P_{\mathrm{rev}}]-k_{1}^{\mathrm{off}}[H_{1}]$$ 5

Writing kinetic equations for the formation of *AA*, *H*_1_, *H*_2_, *AQP*, *H*_1_ *Q*, and *PD* leads to the following system of 12 ODEs (corresponding to the 12 molecular species whose concentrations are recorded at each cycle in *dict_conc*):

$$\frac{d[AA]}{dt}=k_{r}^{\mathrm{on}}[A_{\mathrm{for}}][A_{\mathrm{rev}}]-k_{r}^{\mathrm{off}}[AA]$$ 6

$$\frac{d[A_{\mathrm{for}}]}{dt}=-k_{r}^{\mathrm{on}}[A_{\mathrm{for}}][A_{\mathrm{rev}}]+k_{r}^{\mathrm{off}}[AA]-k_{Q}^{\mathrm{on}}[A_{\mathrm{for}}][Q]+k_{Q}^{\mathrm{off}}[AQ]-k_{1}^{\mathrm{on}}[A_{\mathrm{for}}][P_{\mathrm{rev}}]+k_{1}^{\mathrm{off}}[H_{1}]$$ 7

$$\frac{d[A_{\mathrm{rev}}]}{dt}=-k_{2}^{\mathrm{on}}[A_{\mathrm{rev}}][P_{\mathrm{for}}]+k_{r}^{\mathrm{off}}[AA]-k_{r}^{\mathrm{on}}[A_{\mathrm{for}}][A_{\mathrm{rev}}]+k_{2}^{\mathrm{off}}[H_{2}]$$ 8

$$\frac{d[P_{\mathrm{for}}]}{dt}=-k_{2}^{\mathrm{on}}[A_{\mathrm{rev}}][P_{\mathrm{for}}]+k_{\mathrm{PD}}^{\mathrm{off}}[PD]-k_{\mathrm{PD}}^{\mathrm{on}}[P_{\mathrm{for}}][P_{\mathrm{rev}}]+k_{2}^{\mathrm{off}}[H_{2}]$$ 9

$$\frac{d[P_{\mathrm{rev}}]}{dt}=-k_{1}^{\mathrm{on}}[A_{\mathrm{for}}][P_{\mathrm{rev}}]+k_{\mathrm{PD}}^{\mathrm{off}}[PD]-k_{\mathrm{PD}}^{\mathrm{on}}[P_{\mathrm{for}}][P_{\mathrm{rev}}]+k_{1}^{\mathrm{off}}[H_{1}]-k_{1}^{\mathrm{on}}[AQ][P_{\mathrm{rev}}]+k_{1}^{\mathrm{off}}([AQP]+[H_{1}Q])$$ 10

$$\frac{d[Q]}{dt}=-k_{Q}^{\mathrm{on}}[A_{\mathrm{for}}][Q]+k_{Q}^{\mathrm{off}}[AQ]-k_{Q}^{\mathrm{on}}[H_{1}][Q]+k_{Q}^{\mathrm{off}}([H_{1}Q]+[AQP])$$ 11

$$\frac{d[H_{1}]}{dt}=k_{1}^{\mathrm{on}}[A_{\mathrm{for}}][P_{\mathrm{rev}}]-k_{1}^{\mathrm{off}}[H_{1}]-k_{Q}^{\mathrm{on}}[H_{1}][Q]+k_{Q}^{\mathrm{off}}([H_{1}Q]+[AQP])$$ 12

$$\frac{d\left[AQ\right]}{dt}=k_{Q}^{\mathrm{on}}\left[A_{\mathrm{for}}\right]\left[Q\right]-k_{Q}^{\mathrm{off}}\left[AQ\right]-k_{1}^{\mathrm{on}}\left[AQ\right]\left[P_{\mathrm{rev}}\right]+k_{1}^{\mathrm{off}}\left(\left[AQP\right]+\left[H_{1}Q\right]\right)$$ 13

$$\frac{d[AQP]}{dt}=k_{1}^{\mathrm{on}}[AQ][P_{\mathrm{rev}}]-k_{1}^{\mathrm{off}}[AQP]-k_{Q}^{\mathrm{off}}[AQP]$$ 14

$$\frac{d[H_{1}Q]}{dt}=k_{Q}^{\mathrm{on}}[H_{1}][Q]-k_{Q}^{\mathrm{off}}[H_{1}Q]-k_{1}^{\mathrm{off}}[H_{1}Q]$$ 15

$$\frac{d[H_{2}]}{dt}=k_{2}^{\mathrm{on}}[A_{\mathrm{rev}}][P_{\mathrm{for}}]-k_{2}^{\mathrm{off}}[H_{2}]$$ 16

$$\frac{d[PD]}{dt}=k_{\mathrm{PD}}^{\mathrm{on}}[P_{\mathrm{for}}][P_{\mathrm{rev}}]-k_{\mathrm{PD}}^{\mathrm{off}}[PD]$$ 17

Here, the pair {*k*_1_^on^, *k*_1_^off^} relates to the annealing of *P*_rev_ to *A*_for_, {*k*_2_^on^, *k*_2_^off^} to the annealing of *P*_for_ to *A*_rev_, {*k*_Q_^on^, *k*_Q_^off^} to the annealing of *Q* to *A*_for_, and {*k*_r_^on^, *k*_r_^off^} to the reannealing of *A*_for_ and *A*_rev_ into *AA*. We assume that in the case of reannealing, the free energy accumulated along the length of the double-stranded amplicon would make it virtually impossible for the duplex to dissociate back into *A*_for_ and *A*_rev_ at a temperature equal to *temp_annealing*, and thus set *k*_r_^off^ = 0 (as in Gevertz et al.^13^). For the pair {*k*_PD_^on^, *k*_PD_^off^}, we cannot simply use the simple nearest neighbor thermodynamics formulas embedded in *Biopython* because the nonspecific binding of *P*_for_ and *P*_rev_ into *PD* is not in perfect match. Thus, we used NUPACK^21^ to calculate the free energy associated with the formation of *PD* (Δ*G*_*PD*_), from which we derived estimates of *K*_*PD*_. NUPACK was chosen for its ability to compute complex equilibrium concentrations for arbitrary numbers (including >2) of interacting strand species.

Importantly, even if they represent the same molecular species, the system includes distinct kinetic equations for *AQP* and *H*_1_ *Q*, corresponding to the following reactions:

$$AQ+P_{\mathrm{rev}}\overset{k_{1}^{\mathrm{on}}}{\underset{k_{1}^{\mathrm{off}}}{⇌}}AQP$$ 18

$$H_{1}+Q\overset{k_{Q}^{\mathrm{on}}}{\underset{k_{Q}^{\mathrm{off}}}{⇌}}H_{1}Q$$ 19

Mass conservation is implemented in the system through the following equations:

$$d[A_{\mathrm{for}}]=-(d[AA]+d[H_{1}]+d[AQ]+d[AQP]+d[H_{1}Q])$$ 20

$$d[A_{\mathrm{rev}}]=-(d[AA]+d[H_{2}])$$ 21

$$d[P_{\mathrm{for}}]=-(d[PD]+d[H_{2}])$$ 22

$$d[P_{\mathrm{rev}}]=-(d[PD]+d[H_{1}]+d[AQP]+d[H_{1}Q])$$ 23

$$d[Q]=-(d[AQ]+d[AQP]+d[H_{1}Q])$$ 24

From eq 2, for any given *K*_x_ *,* there are an infinite number of pairs {*k*_x_^on^, *k*_x_^off^}. While the concentrations [*A*_for_]_eq_, [*P*_rev_]_eq,_ and [*H*_1_]_eq_ obtained at equilibrium will directly depend on *K*_*x*_, the speed of the reaction (and hence AC features) will be sensitive to the choice of any pair {*k*_x_^on^, *k*_x_^off^}. Because individual values of *k*_x_^on^ and *k*_x_^off^ cannot easily be estimated for any *x* in any given experimental setting, we assume that the same value of *k* ^on^ can be applied to all molecular species of the system, denote it *k*_oligos_^on^, and use a global optimization algorithm to find its ‘optimal’ value. Here, ‘optimal’ corresponds to the value that best fits experimental data obtained for a given assay (i.e., a specific combination of template, primers, and probe): it minimizes the discrepancy—typically computed as a mean squared error—between predicted and experimental ACs. We then compute individual *k*_x_^off^ values (*k*_1_^off^, *k*_2_^off^, *k*_Q_^off^, *k*_PD_^off^) by using the generic formula $k_{x}^{\mathrm{off}}=\frac{k_{\mathrm{oligos}}^{\mathrm{on}}}{K_{x}}$ Using this strategy, we obtain individual *k* ^off^ values capturing the thermodynamics of binding for each oligo (through the computation of individual Δ*G* and *K* from sequence data) while introducing flexibility in the system because *k* ^on^ is inferred from experimental data for each assay.

This system of 17 kinetic and mass conservation eqs (eqs 6–17 and 20–24) is solved at each cycle by the *solve_ivp* function of the *scipy* package, using values obtained at the end of the denaturation step of the same cycle as initial values, across 100 time steps between *t* = 0 and *t* = *time_annealing* (as specified in *dict_PCR*). We have found that due to the steepness of the problem, the LSODA^22^ or BDF^23^ methods were the most suitable for solving this system, with LSODA having the advantage over BDF of being easily parallelized out-of-the-box with packages such as *concurrent* or *multiprocessing*. As shown in Figure 2, the concentrations of all molecular species can be tracked as a function of time during the annealing phase, and the conservation of mass can be verified graphically. Computing concentrations of oligos during the annealing step is the most time-consuming step of a PCR cycle, but with this implementation, we have found that calling the ***run_PCR*** function over 40–60 cycles gives a result in <5 s. on a standard laptop PC.

**Figure 2:**
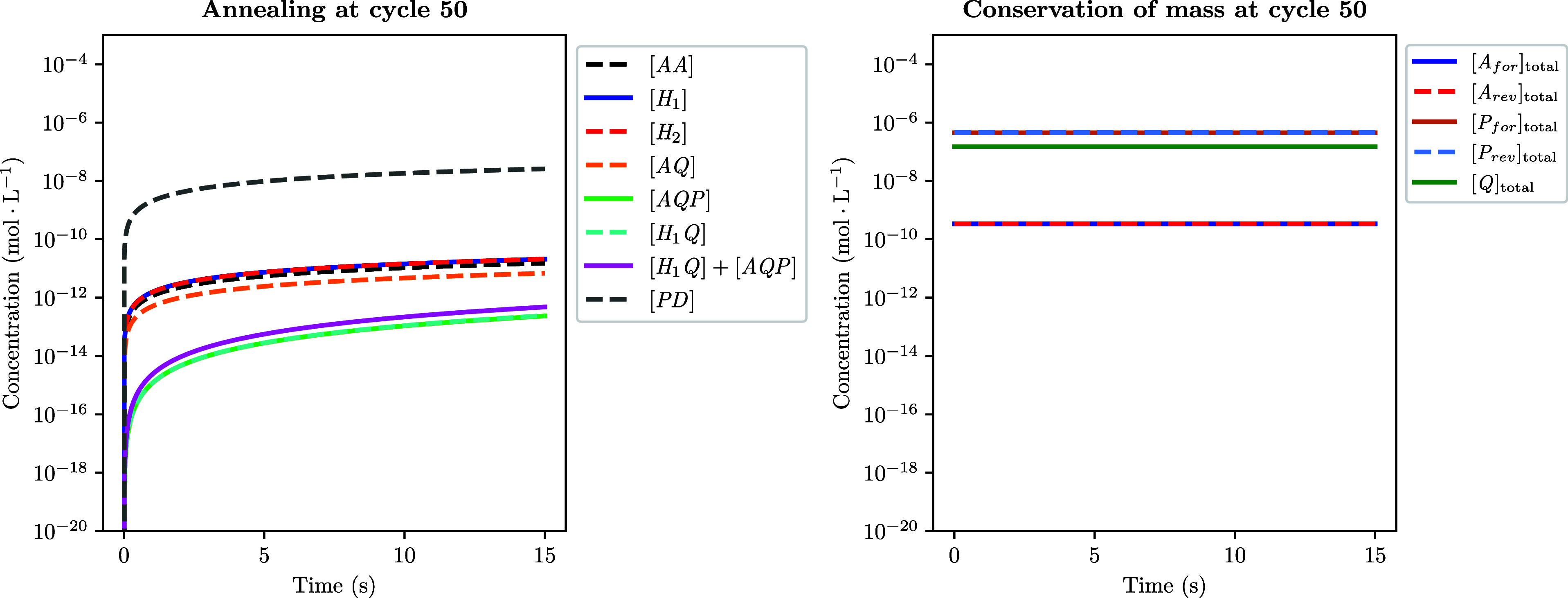
Concentrations of key molecular species and conservation of mass over time during the annealing step of PCR. These plots can be generated when executing the *annealing* function within *run_PCR* in DYNAMIC. The left panel displays the concentrations of key molecular species as a function of time over the duration of the annealing phase. These are computed at each time step by solving a system of 17 kinetic and mass conservation equations that govern molecular interactions during the annealing process. The right panel displays the total concentrations of key molecular species (in their bound and unbound forms) and allows to visually confirm conservation of mass during annealing. This is for instance: [*A*_for_]_total_ = [*AA*]_*t*_ + [*A*_for_]_*t*_ + [*H*_2_]_*t*_, and [*A*_rev_]_total_ = [*AA*]_*t*_ + [*A*_rev_]_*t*_ + [*H*_1_]_*t*_ + [*H*_1_ *Q*]_*t*_ + [*AQP*]_*t*_, where the subscript *t* denotes a concentration at a given time *t*. Note the logarithmic *y*-axis, emphasizing the dynamic range (and stiffness) of the problem. In this simulation, we used the following parameters: number of cycles *N*_cycles_ = 60 and annealing temperature of 60 °C; input double-stranded DNA concentration [*AA*] = 10^7^ copies · μL^–1^; primer concentrations [*P*_for_] = [*P*_rev_] = 450 nmol ·L^–1^; probe concentration [*Q*] = 150 nmol ·L^–1^; hybridization rate constant *k*_on_ = 10^4^ M^–1^ s^–1^ for all oligos (*P*_for_, *P*_rev_, *Q*); reannealing rate constant *k*_r_^on^ = 10^7^ M^–1^ s^–1^; *k*_r_^off^ = 0; *k*_deg_ = 0, β = 1, i.e., no thermal degradation of the Taq polymerase across cycles. We purposely used a low value for *k*_on_—one that does not yield significant amplification over 60 cycles—to offer better visualization of the annealing process over the duration of *time_annealing*. A similar plot obtained with a more realistic value of *k*_on_ = 10^6^ M^–1^ s^–1^ is presented in the Supporting Information.

Finally, *run_PCR* returns a dictionary *dict_conc* where the concentrations of the 12 single-stranded oligos and hybrids are computed over *N*_cycles_ cycles (i.e., a dictionary with 12 key-value pairs, with each item being a one-dimensional vector of length *N*_cycles_). A plotting function displays the evolution of *AA*, *A*_for_, *A*_rev_, *P*_for_, *P*_rev_, and *Q* concentrations across cycles, as well as fluorescence levels (estimated at each cycle during the elongation step from the conversion of *AQP* and *H*_1_ *Q* into *AA*) (see Figure 3).

**Figure 3:**
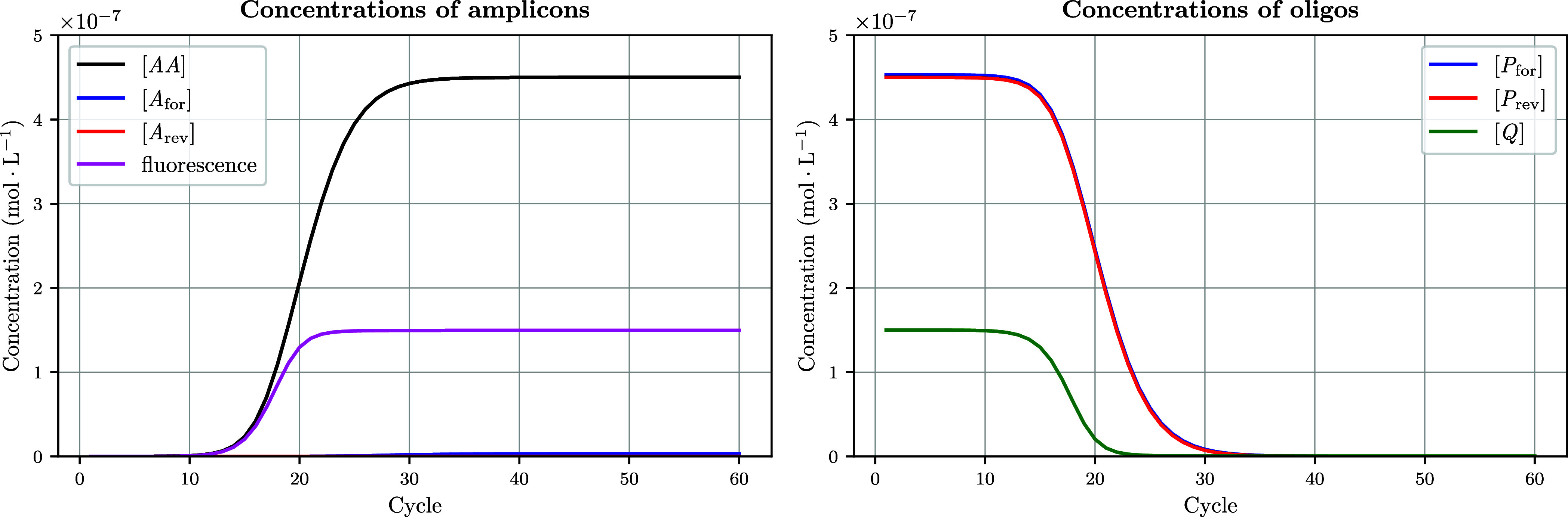
Amplification curve obtained by DYNAMIC, and concentrations of *AA*, *A*_for_, *A*_rev_, *P*_for_, *P*_rev,_ and *Q* across PCR cycles. In this in silico experiment, we ran a real-time TaqMan assay for template C22 (see sequences of the template and oligos in the Supplementary Methods section), with the following parameters: number of cycles *N*_cycles_ = 60 and annealing temperature of 60 °C; input double-stranded DNA concentration [*AA*] = 10^7^ copies·μL^–1^; primer concentrations [*P*_for_] = [*P*_rev_] = 450 nmol·L^–1^; probe concentration [*Q*] = 150 nmol·L^–1^; hybridization rate constant *k*_on_ = 10^6^ M^–1^ s^–1^ for all oligos (*P*_for_, *P*_rev_, *Q*); reannealing rate constant *k*_r_^on^ = 10^7^ M^–1^ s^–1^; *k*_r_^off^ = 0; *k*_deg_ = 0, β = 1, i.e., no thermal degradation of the Taq polymerase across cycles.

#### Flexible Modeling of Taq Thermal Degradation

Even if the Taq polymerase is thermostable, we assume that thermal cycling results in some enzyme deactivation due to the high temperature reached during the denaturation phase of PCR. This impacts the ability of the enzyme to effectively duplicate each DNA molecule at each cycle, predominantly in the later stages of the reaction (as enzyme activity decreases over time). Equivalently, this effect can be interpreted as a decline in PCR efficiency—if the concept of ‘PCR efficiency’ is extended beyond the exponential phase to include later stages of the reaction. Classically, Taq thermal degradation has been modeled through a first-order exponential decay process.^13,14^ However, some of our empirical observations suggested that the classical first-order Taq degradation profile did not provide enough flexibility to accurately model a range of AC features obtained in the dilution series. Thus, we have introduced a more flexible thermal degradation profile, captured in the following 2-parameter decay process:

$${\mathrm{Eff}}_{c}={\mathrm{Eff}}_{0}\cdot \exp(-(k_{\deg}\cdot tim\mathrm{e\_d}enaturation\cdot c{)}^{\beta })$$ 25

where Eff_*c*_ denotes the activity of the Taq polymerase at cycle *c*, Eff_0_ its baseline activity (always set at 1, indicating that at cycle 0 the enzyme has full activity), *k*_deg_ the degradation constant of the enzyme, β a scaling exponent, and *time_denaturation* the duration of the denaturation step at each cycle (in sec.). Of note, when β = 1, the model reduces to a standard first-order decay.

To reflect the impact of enzyme activity on PCR kinetics, we consider that at each cycle, a fraction Eff_c_ of all of the molecular species that could be converted into *AA* during the elongation step is successfully elongated, while the remaining fraction 1 – Eff_c_ remains unconverted due to partial enzyme inactivation. Specifically, at each cycle *c*, we ensure mass conservation of all molecules through the following set of equations:

$$[AA{]}_{c+1}={\mathrm{Eff}}_{c}([H_{1}{]}_{c}+[H_{2}{]}_{c}+[H_{1}Q{]}_{c}+[AQP{]}_{c})+[AA{]}_{c}$$ 26

$${\left[H_{1}\right]}_{c+1}=\left(1-{\mathrm{Eff}}_{c}\right)\cdot {\left[H_{1}\right]}_{c}$$ 27

$${\left[H_{2}\right]}_{c+1}=\left(1-{\mathrm{Eff}}_{c}\right)\cdot {\left[H_{2}\right]}_{c}$$ 28

$${\left[H_{1}Q\right]}_{c+1}=\left(1-{\mathrm{Eff}}_{c}\right)\cdot {\left[H_{1}Q\right]}_{c}$$ 29

$${\left[AQP\right]}_{c+1}=\left(1-{\mathrm{Eff}}_{c}\right)\cdot {\left[AQP\right]}_{c}$$ 30

#### Modeling Serial Dilutions

DYNAMIC provides a function to compute ACs obtained from serial dilutions of the same DNA template. *standard_curve* takes as input a vector of DNA concentrations (typically a standard 10-fold dilution series: 10^7^, 10^6^,..., 1 copies·μL^–1^ + nontemplate control [NTC]); computes PCR simulations and their associated ACs (Figure 4A); extracts cycle threshold (*C*_*t*_) values and returns a plot of *C*_*t*_ values against DNA concentration on a log_10_ scale (Figure 4B). The algorithm is presented below, but in DYNAMIC, this function is accelerated through parallel computing using the *concurrent.futures* module in Python. *C*_*t*_ values are computed using the second-derivative maximum method: for each amplification curve *F*(*c*), where *c* denotes the cycle number, we compute the second derivative *F*″(*c*) with respect to *c*. The *C*_*t*_ is defined as the cycle c* at which *F*″(*c*) reaches its maximum, i.e., the point of maximum acceleration in fluorescence during the exponential phase:

$$c^{*}=\arg\;\max_{c}\;F^{\prime \prime }(c)$$

The same method was used to determine *C*_*t*_ values for experimental and simulated data sets.

**Figure 4:**
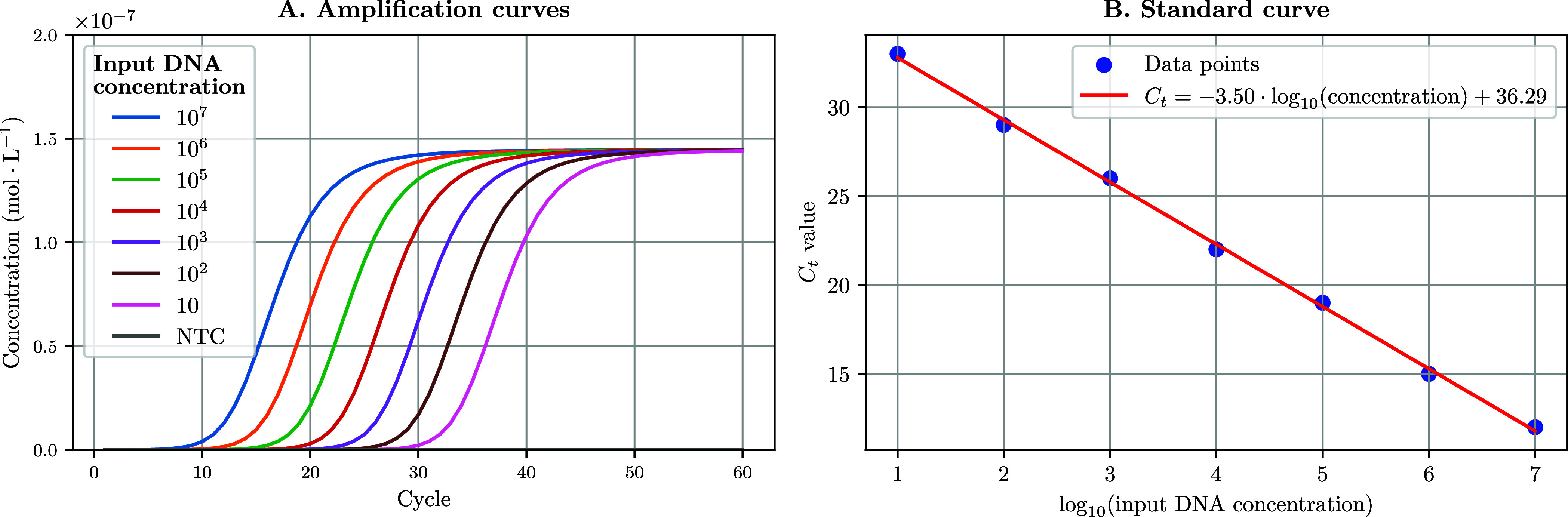
Amplification curves and standard curve obtained by DYNAMIC over a range of 10-fold dilutions. In this in silico experiment, we used the following parameters: *N*_cycles_ = 60, annealing temperature of 60 °C, input double-stranded DNA concentration [*AA*] = 10^7^, 10^6^,..., 1 copies·μL^–1^ + NTC, [*P*_for_] = [*P*_rev_] = 450 nmol·L^–1^, [*Q*] = 150 nmol·L^–1^, *k*_deg_ = 0, *k*_on_ = 10^6^ M^–1^ s^–1^ for all oligos (*P*_for_, *P*_rev_, *Q*), *k*_r_^on^ = 10^7^ M^–1^ s^–1^. *C*_*t*_ values from A are extracted with a custom-made function and plotted against log_10_([DNA]) to obtain a standard curve.

#### Identifying Optimal Hyperparameters To Model Experimental Data

Because we are primarily interested in using DYNAMIC to accurately and precisely model how AC features are influenced by changes in PCR experimental conditions (such as template and oligo sequences and concentrations), we developed an optimization algorithm to identify the set of DYNAMIC hyperparameters that best fits any given experimental data set. Specifically, given an experimental data set *exp_PCR_data* consisting of ACs collected over a range of DNA concentrations for a specific assay (i.e., defined by a particular template, primers, and probes), our goal is to determine the set of hyperparameters args = {*k*_deg_, β, *k*_oligos_^on^, *k*_r_^on^, [DNA]_0_, [*P*_for_]_0_, [*P*_rev_]_0_, [*Q*]_0_} that produces simulated ACs closely matching experimental data.
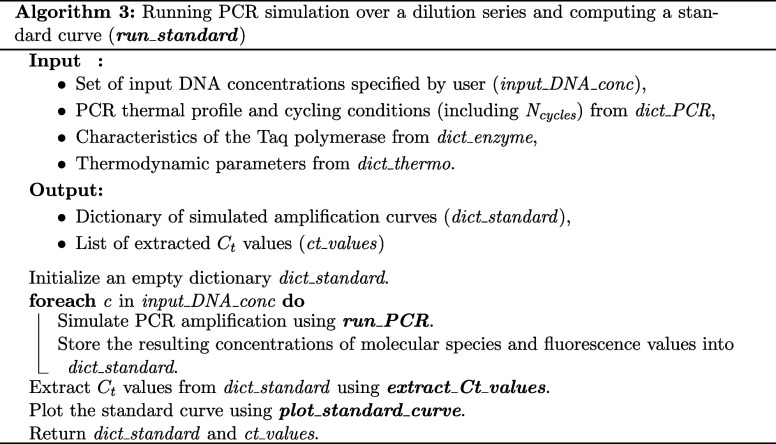


Because the function *run_PCR* is not differentiable with respect to *args*, this task cannot be approached by using gradient-based methods. Instead, it is formulated as a global optimization problem, where multiple candidate solutions are evaluated based on their fitness, defined as the error between *exp_PCR_data* and *run_PCR*(*args). To solve this, we used the differential evolution algorithm implemented in the *optimize* module of the *scipy* package, with a scaled mean squared error (MSE) as the objective function.^24^ The solver was configured with the *best1bin* strategy, a population size of 20, a mutation factor ranging from 0.5 to 1.0, and a recombination constant of 0.7. The convergence tolerance was set to 10^–6^, and the *polish* option was enabled to allow for the final local refinement of the best solution. A detailed pseudoalgorithm is provided below (see Algorithm 4).
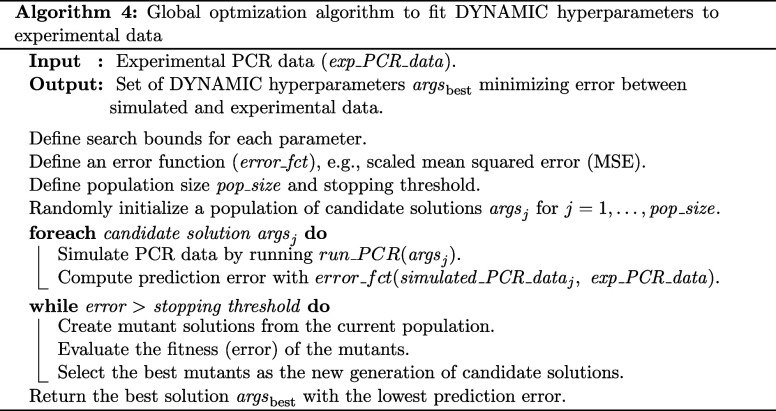


### Experimental Data

#### Assays

We used four assays published previously as part of a 7-plex TaqMan panel for respiratory viruses, targeting the nucleocapsid protein gene (N gene) of human coronaviruses OC43 (HCoV-OC43, denoted COC), HKU1 (HCoV-HKU1, denoted CHK), 229E (HCoV-229E, denoted C22), and Middle East respiratory syndrome-related coronavirus (MERS-CoV, denoted MERS).^12^ Sequences for the DNA templates, primers, and probes of these four targets are presented in the Supporting Information.

#### DNA Templates and Oligos

All primers were synthesized by Integrated DNA Technologies (IDT, United States). TaqMan probes were double-quenched hydrolysis probes labeled with 6-FAM as the fluorophore at the 5*′* end, a ZEN internal quencher, and a 3*′* Iowa Black FQ (3IABkFQ) quencher, also purchased from IDT. In all double-quenched probes, the ZEN moiety was located between the ninth and the 10th nucleotide (in the 5*′* to 3*′* direction). All oligomers were resuspended in TE buffer at a concentration of 100 μmolL^–1^.

Synthetic DNA fragments (gBlocks) were purchased from Integrated DNA Technologies (IDT, United States), resuspended in TE buffer to a concentration of 5 ng/μL, and stored at −20 °C. DNA concentrations were quantified using a NanoDrop One spectrophotometer (ThermoFisher, United States), and serial dilutions were prepared in TE buffer.

#### Experimental Procedures

qPCR mixes contained 5 μL of 2× IDT PrimeTime Gene Expression Master Mix (with low-concentration ROX), 1 μL of 10× primer mix, and 3 μL of DNA templates (or negative control). The final reaction volume was adjusted to 10 μL with nuclease-free water.

All experiments were conducted on a QuantStudio 5 Real-Time PCR System (ThermoFisher, United States). Thermal cycling conditions consisted of a hot-start step at 95 °C for 3 min, followed by 60 cycles at 95 °C for 15 s (denaturation) and 60 °C for 30 to 45 s (annealing and elongation).

The concentrations of oligos used in experiments comparing DYNAMIC predictions to experimental data are presented in the Supporting Information.

### Sigmoidal Fitting

To compare how final fluorescence intensity (FFI, i.e., the plateau of ACs) changes in response to variations in the concentrations of primers and probes in experimental and simulated amplification data, we used a curve-fitting process described in Miglietta et al.^25^ We use the mathematical expression of a 5-parameter sigmoid function relating fluorescence values to cycles^26^:

$$F(c)=F_{b}+\frac{F_{m}}{(1+\exp(S_{c}\times (c-C_{s})){)}^{A_{s}}}$$ 31

where *c* is the PCR cycle number, *F*(*c*) is the fluorescence value obtained at cycle *c*, *F*_*b*_ is the baseline fluorescence value, *F*_*m*_ the FFI, *C*_*s*_ a location parameter indicating the point of inflection, *S*_*c*_ a parameter related to the slope of the linear portion of the curve, and *A*_*s*_ an additional parameter that introduces asymmetry in the overall sigmoid shape.^27^ Curve fitting was performed using the trust region reflective (TRF) algorithm in the *curve_fit* function of the *scipy* package. We then compared *F*_*m*_ values for experimental vs simulated data using linear regression.

## Results and Discussion

### Effects of Concentrations of Probe and Primers on AC Features

First, we sought to assess the accuracy of the DYNAMIC predictions in response to changes in the concentrations of primers and probes. We ran a set of wet lab experiments using the C22 assay, maintaining a constant template concentration [*AA*] = 10^5^ copies·μL^–1^, while varying the concentrations of primers ([*P*_for_] = [*P*_rev_] = 150, 300, or 450 nmol·L^–1^) and probe ([*Q*] = 150, 300, or 450 nmol·L^–1^), resulting in nine different primer mixes. We determined the set of hyperparameters that best fitted the experimental fluorescence data obtained with the ‘classic’ primer mix, corresponding to [*P*_for_] = [*P*_rev_] = 450 nmol·L^–1^ and [*Q*] = 150 nmol·L^–1^, yielding {*k*_deg_ = 1.0 × 10^–4^ s^–1^, β = 1, *k*_oligos_^on^ = 4.5 × 10^5^ M^–1^ s^–1^, *k*_r_^on^ = 10^7^ M^–1^ s^–1^}. Importantly, parameter estimation for oligonucleotide concentrations was not restricted to the exact values used experimentally; rather, only the relative primer-to-probe ratios were enforced. Using these fitted hyperparameters, we then ran DYNAMIC simulations with the remaining eight primer mixes—keeping the thermodynamic hyperparameters fixed and enforcing the same primer-to-probe ratios as in the corresponding experiments—and compared predicted ACs to those obtained experimentally.

The fit between experimental and simulated data can be assessed visually in Figure 5. Although some discrepancies remain, it appears that DYNAMIC accurately captures changes in the PCR kinetics triggered by changes in the primer and probe concentrations. This is especially true for FFI values, which can be compared directly after fitting each AC to a 5-parameter sigmoid function: as presented in Figure S3, the correlation between experimental and simulated FFI values was high (*R* ^2^ = 0.932, *p* < 0.0001). However, while this relationship is clearly linear across the nine primer mixes, only primer-to-probe concentration ratios (not exact concentration values) are preserved across experimental and simulated data, while exact concentration values differ by a factor of ≈1.9. This could be related to a limitation of the software (inaccurate parameter estimation), unmeasured experimental variability, and/or the fact that DYNAMIC does not explicitly model the relationship between end-product concentration and fluorescence levels.

**Figure 5:**
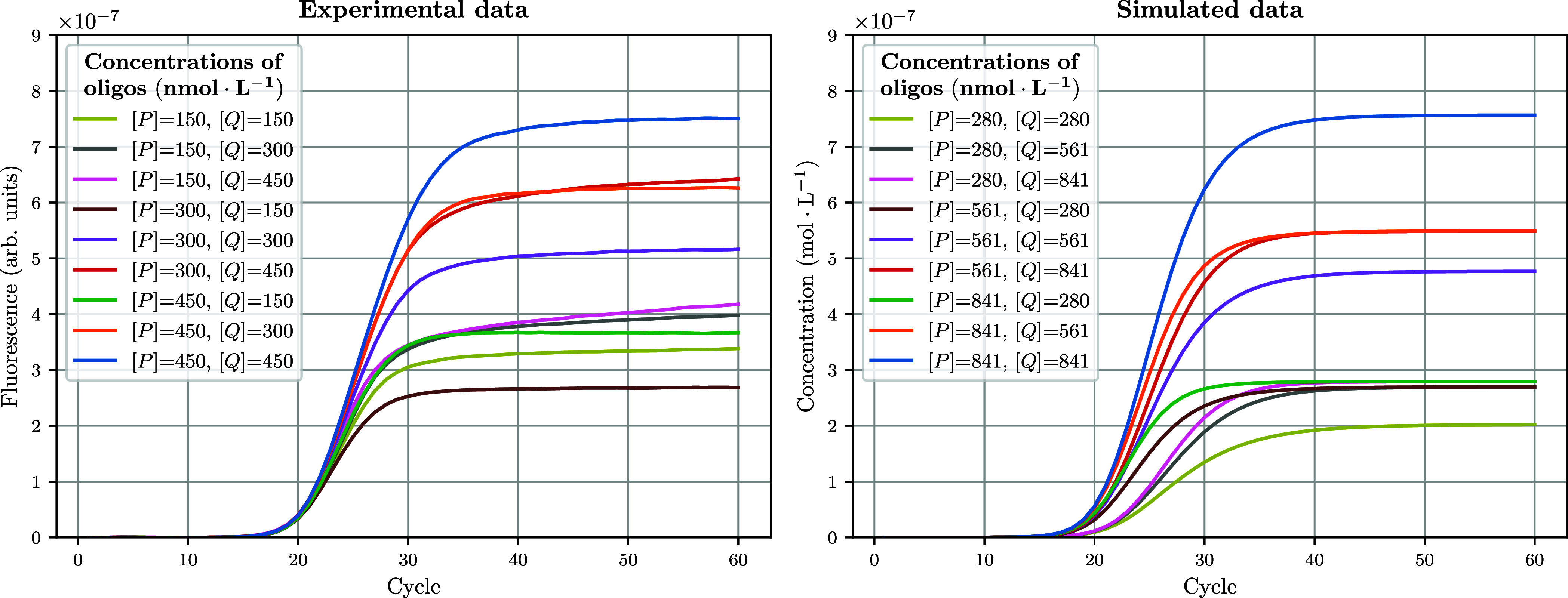
Predictions of amplification curves by DYNAMIC in response to changes in the concentrations of primers and probe. The left panel shows experimental ACs obtained with the C22 template at a concentration of 10^5^ copies·μL^–1^, and varying concentrations of primers and probe. For comparison, the right panel shows ACs obtained through DYNAMIC simulations with a common set of estimated thermodynamic hyperparameters and oligo concentrations constrained to preserve the experimental primer-to-probe ratios for each mix. See also Figure S3, displaying a linear regression plot of simulated vs experimental FFI values.

### Effects of Taq Polymerase Activity

While changes in Taq activity over time—and how these would impact AC features—are difficult to analyze systematically through wet-lab experiments, they can be predicted through numerical simulation. As described above, DYNAMIC models the rate of thermal degradation of the enzyme across PCR cycles by a 2-parameter decay function with degradation constant *k*_deg_ and scaling exponent β. Here, we aimed to analyze how changes in Taq activity over cycles would impact AC features. We ran a set of in silico simulations where we increased the value of *k*_deg_ (while keeping β = 1, equivalent to a simple first-order decay process, for simplicity): in Figure 6, each row corresponds to a different *k*_deg_ value, ranging from 0 (no loss of activity) to 2 × 10^–3^ s^–1^ (rapid decline in efficiency).

**Figure 6:**
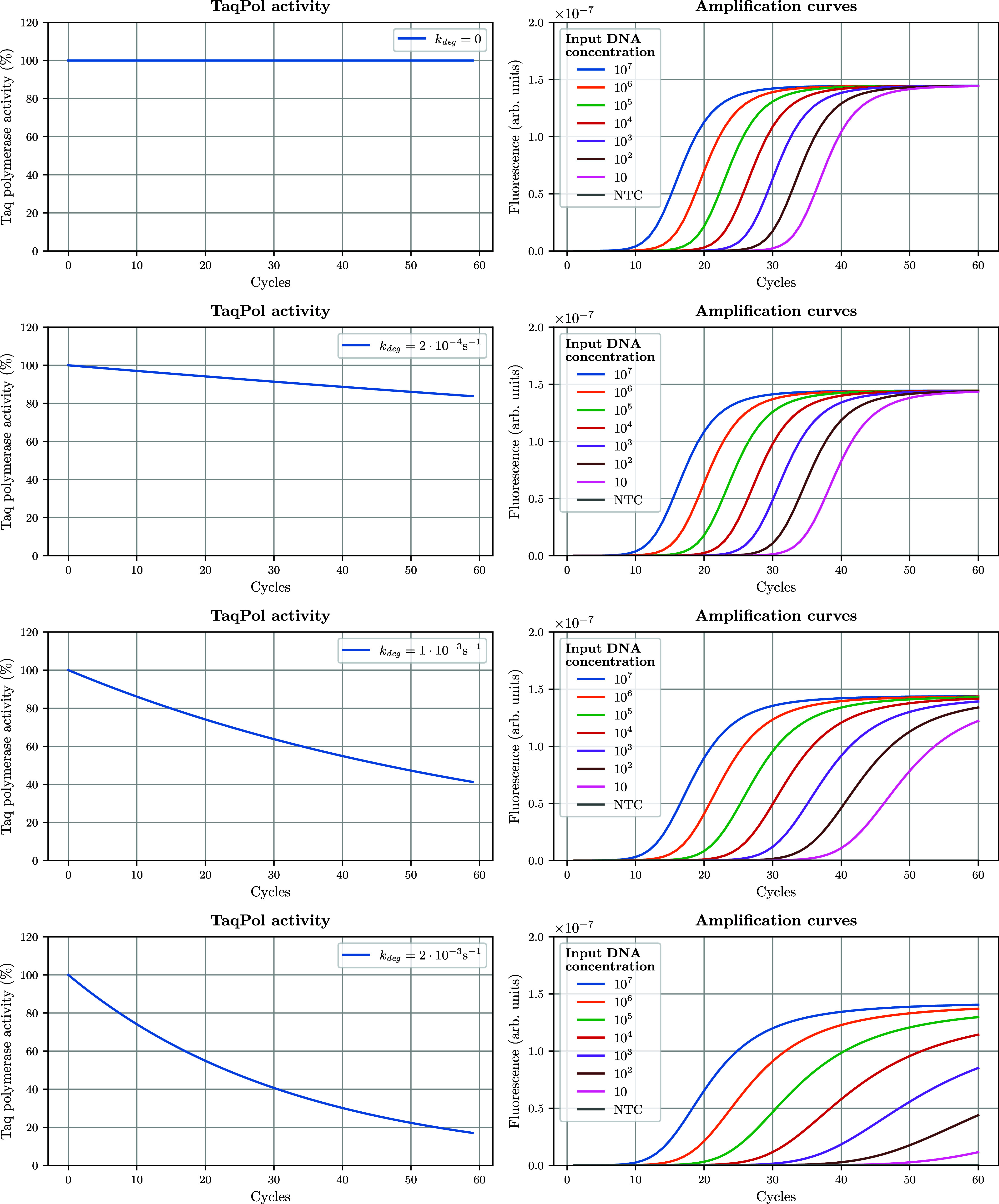
Relationship between Taq polymerase activity across cycles (thermal degradation) and features of amplification curves (as predicted by DYNAMIC). From top to bottom, increasing *k*_deg_ values are associated with increasing thermal degradation of the Taq polymerase across PCR cycles, which impacts AC features. For simplicity here, the second parameter β governing the Taq activity decay is kept at 1 (equivalent to a first-order decay profile). Supplementary Figure S4 displays standard curves associated with these four enzymatic degradation profiles and confirms the overall loss of PCR efficiency associated with higher values of *k*_deg_.

When *k*_deg_ = 0, polymerase activity remains constant at 100% throughout the 60 cycles, resulting in early *C*_*t*_ values and steep AC slopes indicative of high PCR efficiency. As *k*_deg_ increases, we observe a progressive decline in polymerase activity over cycles, following first-order kinetics (because β is kept constant at 1; left panels). At most, when *k*_deg_ = 2 × 10^–4^ s^–1^, the Taq has virtually no residual activity after cycle 50, and there is almost no amplification at a template concentration <10^4^ copies·μL^–1^ (or at least it is not detectable on the chosen *y*-axis scale). This also comes with changes in the shapes of ACs, evidenced in the right panel plots: *C*_*t*_ values are not modified for high concentrations of input DNA (i.e., 10^7^, 10^6^ copies·μL^–1^), but *C*_*t*_ values are delayed at lower DNA concentrations (see also Figure S4). Maybe more importantly (and as suggested by the observation described above), thermal degradation strongly impacts the latter portion of ACs, with decreased slopes in the linear portion of the curve and, for the highest *k*_deg_ value, a lower plateau.

### Effects of Association Constants of Oligos and Reannealing

As explained above, association and dissociation constants for oligos (*k*_oligos_^on^ and *k*_oligos_^off^) cannot be calculated precisely. In DYNAMIC, we set a fixed *k*_oligos_^on^ for all simulations, but its exact value needs to be selected by the software user. To investigate how *k*_oligos_^on^ values impact AC shapes, we conducted a series of in silico experiments, presented in Figure 7, where we investigate how ACs obtained across a dilution series are modified with values of *k*_r_^on^ increasing from 2 × 10^5^ to 10^6^ M^–1^ s^–1^ (left to right panels).

**Figure 7:**
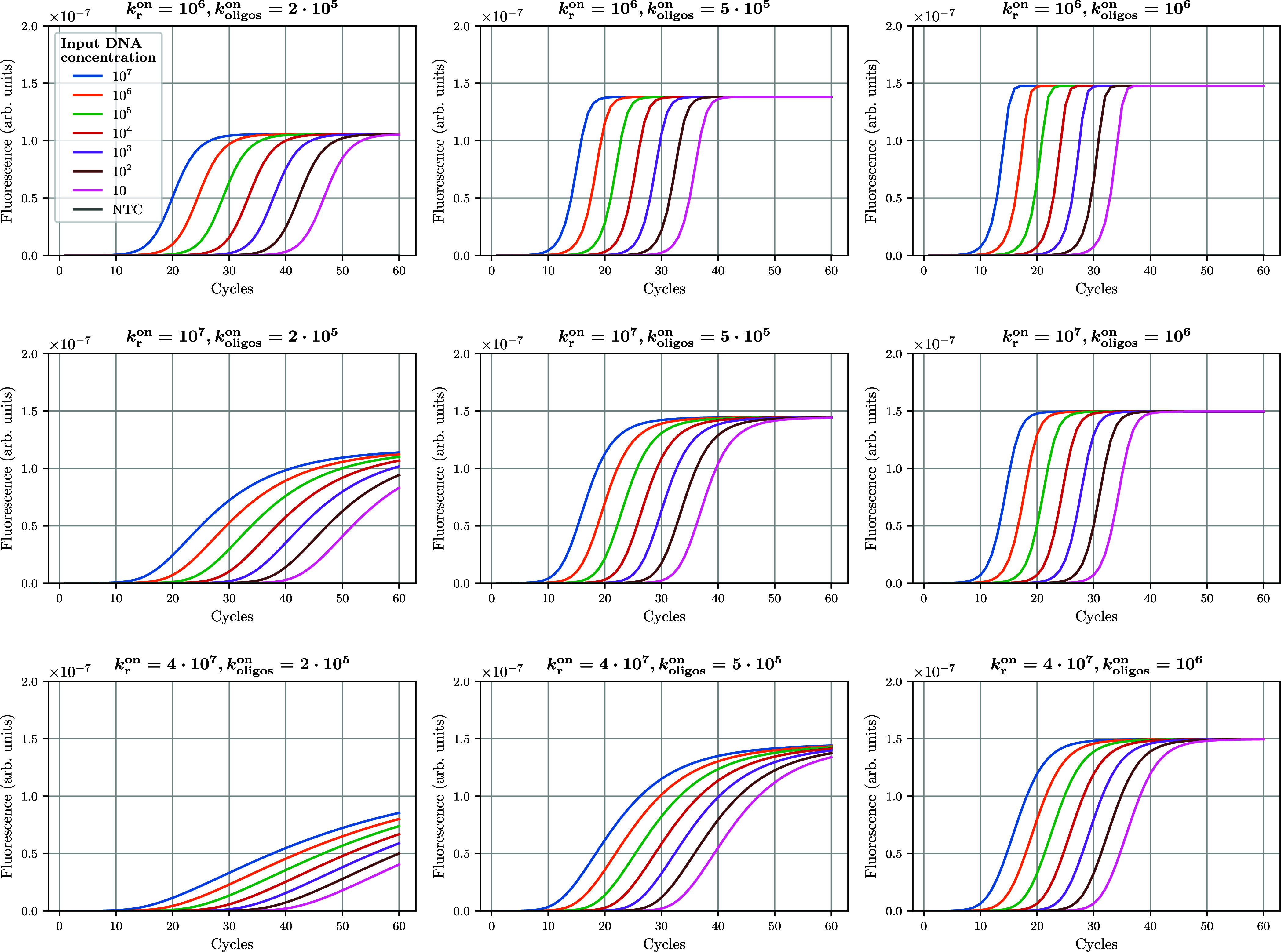
Effect of changes in association constants *k*_oligos_^on^ (corresponding to the annealing of oligos *P*_for_, *P*_rev_ and *Q* on their corresponding template strand) and reannealing constant *k*_r_^on^ on AC features. Units for *k*_r_^on^ and *k*_oligos_^on^ are M^–1^ s^–1^.

Empirical observations suggested that the reannealing of *A*_for_ and *A*_rev_ into *AA* also had a strong impact on AC features. This reaction can be described as

$$A_{\mathrm{for}}+A_{\mathrm{rev}}\overset{k_{r}^{\mathrm{on}}}{\underset{k_{r}^{\mathrm{off}}}{⇌}}AA$$ 32

and while we set *k*_r_^off^ = 0, the exact value of *k*_r_^on^ is unknown. Thus, Figure 7 also explores how increasing values of *k*_r_^on^ from 10^6^ to 4 × 10^7^ M^–1^ s^–1^ (top to bottom panels), associated with more reannealing, impact AC features.

During amplification, the effects of *k*_oligos_^on^ and *k*_*r*_^on^ on amplification kinetics are opposite: PCR efficiency at later cycles (mostly evidenced by changes in the slope) increases with higher values of *k*_oligos_^on^, but decreases with higher values of *k*_r_^on^, reflecting the competition between reaction 32 and the formation of hybrids *H*_1_, *H*_2_, *AQP*, and *H*_1_ *Q* from *A*_for_ and *A*_rev_. Interestingly, the matrix of AC simulations is not strictly symmetrical, suggesting some inherent complexity (i.e., nonredundancy) in the effect of *k*_oligos_^on^ and *k*_r_^on^ on AC features.

### Fitting DYNAMIC Hyperparameters to Experimental Data

We have shown that DYNAMIC offers accurate insights into how changes in experimental conditions and thermodynamic parameters influence AC features. However, the complexity and inherent nonredundancy in these relationships have suggested that several distinct values for the set of DYNAMIC hyperparameters could yield ACs with similar features. This has motivated the development of a global optimization algorithm (Algorithm 4) that automatically identifies optimal sets of hyperparameters to fit any given experimental data set. Figure 8 provides two examples of results generated by our fitting algorithm, applied to experimental dilution series obtained with the CHK and COC templates: the discrepancy between simulated and experimental data appears minimal, indicating an excellent fit (averaged scaled MSE 0.074 ± 0.022 and 0.101 ± 0.002, respectively). Two similar examples (for targets C22 and MERS) are presented in Supplementary Figure S5.

**Figure 8:**
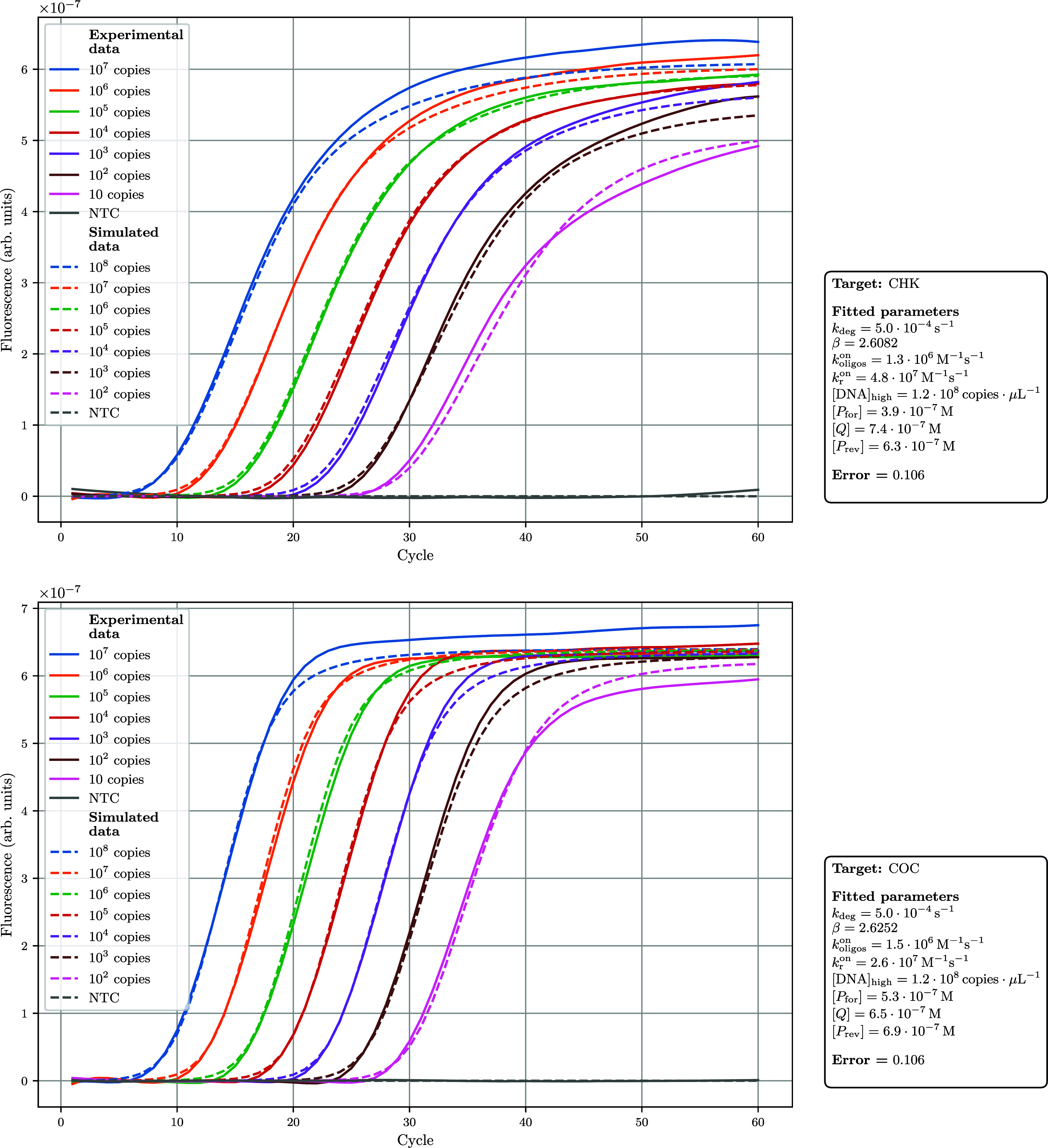
Simulated and experimental amplification curves across a DNA dilution series for the CHK and COC templates. Experimental amplification curves (solid lines) are shown alongside simulated curves (dashed lines) for a dilution series of the CHK and COC targets. Parameters were fitted using the global optimization algorithm within DYNAMIC. Taq efficiency profile over cycles was modeled through a 2-parameter decay process. The close overlap between experimental and predicted curves illustrates the model’s ability to accurately capture amplification kinetics across DNA concentrations. Averaged scaled MSE 0.074 ± 0.022 and 0.101 ± 0.002, respectively.

Notably—and consistent with our intuition—running the same fitting process multiple times can yield different sets of hyperparameters, yet the predicted ACs remain nearly indistinguishable. For instance, with the CHK template, the parameter set shown in Figure 8 (*k*_deg_ = 5.0 × 10^–4^ s^–1^, β = 2.61, *k*_oligos_^on^ = 1.3 × 10^5^ M^–1^ s^–1^, *k*_r_^on^ = 4.8 × 10^7^ M^–1^ s^–1^, [DNA]_high_ = 1.2 × 10^8^ copies μL^–1^, [*P*_for_] = 3.9 × 10^–7^ mol·L^–1^, [*Q*] = 7.4 × 10^–7^ mol·L^–1^, [*P*_rev_] = 6.3 × 10^–7^ mol·L^–1^) produces ACs visually similar to those generated by other parameter sets (data not shown).

Finally, the global optimization algorithm offers interesting insights into the relationship between Taq activity profile over cycles and AC features. When setting β = 1 (i.e., when Taq thermal degradation follows strict first-order kinetics) and running our fitting algorithm with identical parameters (including bounds for DYNAMIC thermodynamic hyperparameters and stopping criteria), we observe a decreased flexibility to model experimental ACs over the four targets from the 7-plex panel. This is evidenced visually because the discrepancy between predicted and experimental ACs appears higher, and confirmed mathematically through significantly higher MSE values (see Supplementary Figures S6 and S7).

### Limitations and Future Work

Some authors have developed models describing the incorporation of deoxynucleotide triphosphates (dNTPs) during the elongation phase of PCR.^17^ These models are particularly valuable as they can precisely characterize the population of incomplete amplicons (i.e., truncated at the 3*′* end) that arise when the duration of elongation is short relative to the polymerization speed of the Taq polymerase.^28^ In assays using intercalating dyes, these truncated *A*_for_ and *A*_rev_ molecules may still generate fluorescence as the dyes bind to the double-stranded regions of hybrid structures formed between full-length strands and their truncated reverse complements. However, in subsequent cycles, if the 3*′* end of a truncated single-stranded amplicon falls upstream of the primer binding site, further elongation becomes impossible, resulting in incomplete amplification. This phenomenon gives rise to ACs with characteristically low slopes—often approaching linearity. Although this behavior has not yet been modeled in DYNAMIC, it represents a potential avenue for future development.

It has been reported that the *Taq* polymerase does not exhibit a strong preference between single-stranded and double-stranded DNA and can bind nonspecifically to multiple DNA sites.^17^ This nonspecific binding can lead to the formation of *Taq*–DNA complexes that are unsuitable for further elongation, ultimately impeding the amplification of double-stranded amplicons over successive PCR cycles. Longer DNA species are expected to bind more polymerase molecules, increasing the likelihood of such an inhibitory complex formation. Inspired by previous work,^17^ we implemented in DYNAMIC a specific kinetic model of *Taq* polymerase binding to DNA. Our goal was to characterize as precisely as possible the loss of polymerase activity in the later cycles of PCR (beyond the cycle threshold), in order to accurately model amplification kinetics and reproduce experimental ACs that exhibit a clear exponential phase (i.e., a well-defined *C*_*t*_) but a reduced slope and FFI. However, incorporating a mechanistic model of nonspecific *Taq*–DNA binding requires solving a system of ODEs akin to those used for modeling oligo and amplicon hybridization. This approach proved prohibitively time- and resource-intensive. Instead, we found that a flexible model of *Taq* thermal degradation based on a 2-parameter decay formula (i.e., more complex than the first-order kinetics classically used to describe Taq thermal degradation) was sufficient to capture a range of PCR efficiency profiles and to generate in silico ACs that closely resembled experimental data. As a result, the more detailed model of nonspecific *Taq* binding was not used in our final analyses.

Despite its flexibility and interesting predictive capabilities, DYNAMIC remains a simplified representation of the molecular events underlying single-plex PCR and presents several important limitations. As evidenced in the experiments presented in Figures 8 and S5–S7, the fit between experimental data and DYNAMIC predictions of standard dilution series varies between assays, with, for instance, a better overall fit for the CHK (Figure 8) than for the C22 assay (Figure S5), and between ACs corresponding to different concentrations of input DNA for a given assay. This could be explained by several factors. First, the model could lack the flexibility to capture subtle variations in experimental conditions. Second, DYNAMIC assumes ideal reaction conditions and does not account for experimental variability arising from batch effects, pipetting errors, or the presence of inhibitors in clinical samples—all of which can affect amplification efficiency and AC morphology. Third, DYNAMIC estimates association and dissociation constants for oligonucleotides using published thermodynamic parameters derived from the nearest-neighbor model of nucleic acid interactions. While this approach provides a reasonable approximation, it does not account for more complex biophysical phenomena, such as the formation of secondary structures (e.g., hairpins, G-quadruplexes) or off-target hybridization events. Although more sophisticated thermodynamic models—such as those implemented in NUPACK^21^—have been developed to address these limitations, all existing models are subject to inherent inaccuracies, and substantial discrepancies persist between the predicted behavior of oligos and experimental results. Fourth, while primer–dimer formation between forward and reverse primers (PD) is included in DYNAMIC, other potential nonspecific interactions (e.g., primer–probe dimers or more extensive primer self-dimerization beyond the defined PD) are not considered in the current reaction network.

Finally, DYNAMIC is designed and optimized as a singleplex PCR simulator; extending its capabilities to accurately model multiplex PCR would require substantial additions to account for interassay competition for reagents and more complex oligo interactions. While the software supports parallel computing and is capable of running simulations in an acceptable time frame for most applications, runtime remains a bottleneck for large-scale inference (i.e., to use it, for instance, for data augmentation purposes in ML applications).

## Conclusions

In this article, we describe DYNAMIC, an open-source Python implementation of a kinetic model of PCR based on established kinetic and stoichiometric principles. By explicitly and separately modeling the annealing behavior of primers and probe, incorporating primer–dimer formation and amplicon reannealing, and introducing a flexible two-parameter model of Taq activity decay, DYNAMIC reproduces key aspects of experimental kinetics, including shifts in *C*_*t*_, changes in slope during late amplification, and variations in FFI in response to oligo concentrations. Coupled to a global optimization routine, the framework robustly fits assay-specific thermodynamic and kinetic hyperparameters from dilution-series data across multiple published targets, enabling accurate in silico prediction of full AC shapes rather than only early cycle amplification kinetics.

Overall, DYNAMIC provides a practical digital twin of real-time singleplex TaqMan PCR that can reduce empirical trial-and-error during assay development and support rational “tailored chemistry” strategies for ACA. Future extensions will focus on incorporating additional sources of nonideal behavior (e.g., secondary structures, inhibitors, truncated products), improving the identifiability of fitted parameters, and scaling toward multiplex settings where interassay competition and richer oligo interaction networks can impact AC morphology.

## Supplementary Material



## Notes

Supplementary Methods: accession numbers, characteristics, sequences, and melting temperatures of DNA templates, primers, and probes for the four targets from the 7-plex TaqMan panel for respiratory viruses; concentrations of primers and probes used in experiments comparing experimental and simulated ACs; Supplementary Figure S1: concentrations of key molecular species and conservation of mass over time during the annealing step of PCR with realistic hybridization rate constant; Supplementary Figure S2: simulated ACs and concentrations of molecular species across PCR cycles obtained with atypical (including asymmetric) PCR designs; Supplementary Figure S3: linear regression plot comparing FFI values between experimental and simulated data across nine primer mixes with varying concentrations of primers and probe; Supplementary Figure S4: standard curves predicted by DYNAMIC showing the relationship between Taq polymerase thermal degradation and overall PCR efficiency; and Supplementary Figures S5–S8: simulated and experimental ACs across DNA dilution series for C22, MERS, CHK, and COC templates, comparing two-parameter and first-order Taq activity decay models (PDF)

## Abbreviations

AA: double-stranded amplicon
AC: amplification curve
ACA: amplification curve analysis
AI: artificial intelligence
*AQP*: *A*_for_ + *Q* + *P*_rev_ hybrid
*A*_for_: amplicon forward strand
*A*_rev_: amplicon reverse strand
*A*_*s*_: a parameter that introduces asymmetry in the 5-parameter sigmoid function
CHK: HCoV-HKU1
COC: HCoV-OC43
*C*_*s*_: location parameter at which fluorescence equals *F*_*m*_/2
*C*_*t*_: cycle threshold
DNA: deoxyribonucleic acid
dNTPs: deoxynucleotide triphosphates
FAM: fluorescein
FFI: final fluorescence intensity
*H*_1_: *A*_for_ + *P*_rev_ hybrid
*H*_1_ *Q*: *A*_for_ + *Q* + *P*_rev_ hybrid (formed by annealing of *Q* on *H*_1_)
*H*_2_: *A*_rev_ + *P*_for_ hybrid
IDT: Integrated DNA Technologies
*k*_1_^on^: association constant for the annealing of *P*_rev_ on *A*_for_
*k*_1_^off^: dissociation constant for the annealing of *P*_rev_ on *A*_for_
*k*_2_^on^: association constant for the annealing of *P*_for_ on *A*_rev_
*k*_2_^off^: dissociation constant for the annealing of *P*_for_ on *A*_rev_
*k*_Q_^on^: association constant for the annealing of *Q* on *A*_for_
*k*_Q_^off^: dissociation constant for the annealing of *Q* on *A*_for_
*k*_r_^on^: association constant for the reannealing of *A*_for_ and *A*_rev_ into *AA*
*k*_r_^off^: dissociation constant for the reannealing of *A*_for_ and *A*_rev_ into *AA*
*k*_PD_^on^: association constant for primer dimer formation
*k*_PD_^off^: dissociation constant for primer dimer dissociation
*k*_deg_: thermo-degradation constant of the enzyme
MC: melting curve
MCA: melting curve analysis
MERS-CoV: Middle East Respiratory Syndrome-related Coronavirus
ML: machine learning
mPCR: multiplex PCR
N gene: nucleocapsid protein gene
NTC: Non-Template Control
ODE: Ordinary Differential Equations
*P*_for_: forward primer
*P*_rev_: reverse primer
PCR: polymerase chain reaction
*PD*: primer dimer
*Q*: TaqMan probe
qPCR: real-time PCR
RNA: ribonucleic acid
*S*_*c*_: parameter related to the slope of the linear portion of the 5-parameter sigmoid
*T*: temperature (in Kelvin)
*T*_m_: melting temperature

## References

[1] Kreitmann L. (2023). Next-generation molecular
diagnostics: Leveraging digital technologies
to enhance multiplexing in real-time PCR. TrAC,
Trends Anal. Chem..

[2] Holma T. (2022). Rapid molecular detection
of pathogenic microorganisms and antimicrobial
resistance markers in blood cultures: evaluation and utility of the
next-generation FilmArray Blood Culture Identification 2 panel. Eur. J. Clin. Microbiol. Infect. Dis..

[3] Kreitmann L., Gaudet A., Voiriot G. (2025). PCR multiplex
en réanimation. Intens. Care Med..

[4] Wittwer C. T. (2001). Real-Time Multiplex PCR Assays. Methods.

[5] Wittwer C. T. (2009). High-resolution
DNA melting analysis: advancements and limitations. Human Mutation.

[6] Montgomery J. L., Sanford L. N., Wittwer C. T. (2010). High-resolution DNA melting analysis
in clinical research and diagnostics. Expert
Rev. Mol. Diagn..

[7] Holland P. M. (1991). Detection of specific polymerase chain reaction product by utilizing
the 5′—-3′ exonuclease activity of Thermus aquaticus
DNA polymerase. Proc. Natl. Acad. Sci. U. S.
A..

[8] Moniri A. (2020). Amplification Curve
Analysis: Data-Driven Multiplexing Using Real-Time
Digital PCR. Anal. Chem..

[9] Moniri A. (2020). High-Level Multiplexing
in Digital PCR with Intercalating Dyes by
Coupling Real-Time Kinetics and Melting Curve Analysis. Anal. Chem..

[10] Miglietta L. (2021). Coupling Machine Learning and High Throughput
Multiplex Digital PCR
Enables Accurate Detection of Carbapenem-Resistant Genes in Clinical
Isolates. Front. Mol. Biosci..

[11] Mao Y. (2023). Deep Domain Adaptation Enhances Amplification Curve
Analysis for
Single-Channel Multiplexing in Real-Time PCR. IEEE J. Biomed. Health Informat..

[12] Miglietta L. (2023). Smart-Plexer: a breakthrough workflow for hybrid
development of multiplex
PCR assays. Commun. Biol..

[13] Gevertz J. L., Dunn S. M., Roth C. M. (2005). Mathematical model
of real-time PCR
kinetics. Biotechnol. Bioeng..

[14] Mehra S., Hu W.-S. (2005). A kinetic model
of quantitative real-time polymerase chain reaction. Biotechnol. Bioeng..

[15] Rutledge R. G., Côté C. (2003). Mathematics of quantitative kinetic PCR and the application
of standard curves. Nucleic Acids Res..

[16] Smith M. V. (2007). Absolute estimation
of initial concentrations of amplicon in a real-time
RT-PCR process. BMC Bioinformat..

[17] Tafur, D. Novel Kinetic Description of Real-Time Polymerase Chain Reaction Characterizes Interrelated Effects of Sample, Master Mix, and Cycle Time. PhD Thesis, Utah State University, 2024.

[18] SantaLucia, J. Physical Principles and Visual-OMP Software for Optimal PCR Design. In PCR Primer Design; Yuryev, Y. ; Totowa, N. J. , Eds.; Humana Press, 2007; pp 3–33. 10.1007/978-1-59745-528-2_1 17951788

[19] Booth C. S. (2010). Efficiency of the Polymerase
Chain Reaction. Chem. Eng. Sci..

[20] Millington A. L. (2019). The kinetic requirements
of extreme qPCR. Biomol.
Detect. Quantificat..

[21] Fornace M. E., Porubsky N. J., Pierce N. A. (2020). A Unified
Dynamic Programming Framework
for the Analysis of Interacting Nucleic Acid Strands: Enhanced Models,
Scalability, and Speed. ACS Synth. Biol..

[22] Petzold L. (1983). Automatic
Selection of Methods for Solving Stiff and Nonstiff Systems of Ordinary
Differential Equations. SIAM J. Sci. Stat. Comput..

[23] Byrne G. D., Hindmarsh A. C. (1975). A Polyalgorithm
for the Numerical Solution of Ordinary
Differential Equations. ACM Trans. Math. Softw..

[24] Storn R., Price K. (1997). Differential Evolution
– A Simple and Efficient Heuristic
for global Optimization over Continuous Spaces. J. Global Optimiz..

[25] Miglietta L. (2022). Adaptive Filtering Framework to Remove Nonspecific and Low-Efficiency
Reactions in Multiplex Digital PCR Based on Sigmoidal Trends. Anal. Chem..

[26] Swillens S., Dessars B., El Housni H. (2008). Revisiting
the sigmoidal curve fitting
applied to quantitative real-time PCR data. Anal. Biochem..

[27] Spiess A.-N., Feig C., Ritz C. (2008). Highly accurate sigmoidal fitting
of real-time PCR data by introducing a parameter for asymmetry. BMC Bioinformatics.

[28] Debode F. (2017). The influence of amplicon
length on real-time PCR results. BASE.

