# Supplementary material for: DYNAMIC: A Novel Software Implementation of a Kinetic Model of TaqMan PCR
Source: Anal Chem. 2026 Mar 16;98(12):9089–101. doi: 10.1021/acs.analchem.5c07375 (PMC13044887; doi:10.1021/acs.analchem.5c07375)
Supplement: Supplementary file 1 [file ac5c07375_si_001.pdf]

Supporting Information for Publication

DYNAMIC: a novel software implementation of a kinetic model of TaqMan PCR

Authors
Louis Kreitmann[1,2], Ye Mao[3], Ke Xu[1,3], Alison Holmes[1,4], Karen Brengel-Pesce[5], Laurent Drazek[6,§], and Jesus Rodriguez-Manzano*[1,4,§]

Authors' affiliations
[1]Department of Infectious Disease, Imperial College London, London W12 0NN, United Kingdom
[2]Open Innovation & Partnerships, bioMérieux, 376 Chemin de l'Orme, Marcy-l'Étoile 69280, France
[3]Department of Electrical and Electronic Engineering, Imperial College London, London W12 0NN, United Kingdom
[4]The Fleming Initiative, Imperial College London and Imperial College Healthcare NHS Trust, London W12 0NN, United Kingdom
[5]Molecular Biology, Research & Development, bioMérieux, 5 Rue des Berges, Grenoble 38000, France
[6]Data Science, Research & Development, bioMérieux, 5 Rue des Berges, Grenoble 38000, France
[§]L.D. and J.R.-M. contributed equally to this work as senior authors.
*Email: j.rodriguez-manzano@imperial.ac.uk

Number of pages: 11
Number of figures: 7
Number of tables: 3

Table of Contents
Supplementary Methods: Accession numbers, characteristics, sequences, and melting temperatures of DNA templates, primers, and probes for the four targets from the 7-plex TaqMan panel for respiratory viruses; concentrations of primers and probes used in experiments comparing experimental and simulated ACs.
Supplementary Figure S1: Concentrations of key molecular species and conservation of mass over time during the annealing step of PCR with realistic hybridization rate constant.
Supplementary Figure S2: Simulated ACs and concentrations of molecular species across PCR cycles obtained with atypical (including asymmetric) PCR designs.
Supplementary Figure S3: Linear regression plot comparing FFI values between experimental and simulated data across nine primer mixes with varying concentrations of primers and probe.
Supplementary Figure S4: Standard curves predicted by DYNAMIC showing the relationship between Taq polymerase thermal degradation and overall PCR efficiency.

Supplementary Figures S5–S8: Simulated and experimental ACs across DNA dilution series for C22, MERS, CHK, and COC templates, comparing two-parameter and first-order Taq activity decay models.

Supplementary Methods

Accession numbers and characteristics of the DNA templates of 4 targets from the 7-plex respiratory viruses TaqMan panel are presented in Table S1.

Microorganism	Abbreviation	Gene	Accession No.	Template length (bp)	Amplicon length (bp)
HCoV-229E	C22	N	NC_002645	570	158
HCoV-HKU1	CHK	N	NC_006577	300	206
HCoV-OC43	COC	N	NC_006213	300	115
MERS-CoV	MERS	N	NC_019843	300	67

Supplementary Table S1: **Synthetic DNA templates for the four targets from the 7-plex TaqMan panel for respiratory viruses.**

Sequences and T_m of the primers and probes of the 7-plex respiratory viruses TaqMan panel are presented in Table S2.

Microorganism	Oligo type	Oligo sequence (5′-3′)	Oligo T_m (°C)
C22	forward	GAAATGCAAAAGCCACGGTGGAA	68.67
	probe	AGTTGTGGTCAAGGTCTCTGGGGCC	71.21
	reverse	AGCTCAGCAAATTGTGGATAGCC	67.37
CHK	forward	TCAAGAAGCTATCCCTACTAGGT	63.71
	probe	CGCCTGGTACGATTTTGCCTCAAGGCT	72.21
	reverse	GCGATCTCATCAGCCATATCAGGT	68.29
COC	forward	GGTGGAGAAATGTTAAAACTTGGAACT	66.26
	probe	TCCCCATTCTTGCAGAACTCGCACCCA	73.13
	reverse	CCAAAGAAAAACGCACCAGCTG	66.94
MERS	forward	ACGCGGAACCCTAACAATGATT	66.51
	probe	TTCCCTGGAGGTCTCCTGGTCCGC	72.33
	reverse	GTGGGTCCTCAGTGCCGAGT	69.98

Supplementary Table S2: **Primers and probes for the four targets from the 7-plex TaqMan panel for respiratory viruses.**
TaqMan probes were doubled-quenched hydrolysis probes labelled with 6-FAM as the fluorophore at the 5′ end, a ZEN internal quencher, and a 3′ Iowa Black® FQ (3IABkFQ) quencher, also purchased from IDT. In all doubled-quenched probes, the ZEN moiety was located between the 9th and the 10th nucleotide (in the 5′ to 3′ direction). For instance for the C22 target, the exact sequence was 5′-/56-FAM/AGTTGTGGT/ZEN/CAAGGTCTCTGGGGCC/3IABkFQ/-3′.

Concentrations of primers and probes used in the experiment presented in Figure 5 are shown in Table S3.

Primer mix	$[\mathbf{P}_{\mathrm{for}}]$ (nmol·L^{-1})	[Q] (nmol·L^{-1})	$[\mathbf{P}_{\mathrm{rev}}]$ (nmol·L^{-1})
PM1	150	150	150
PM2	150	300	150
PM3	150	450	150
PM4	300	150	300
PM5	300	300	300
PM6	300	450	300
PM7	450	150	450
PM8	450	300	450
PM9	450	450	450

Supplementary Table S3: **Composition of the nine primer mixes used in experiments comparing experimental and simulated ACs in response to changes in oligo concentrations.**

In experiments presented in Figures 8, S5, S6 and S7, we used primer concentrations $[P_{\mathrm{for}}] = [P_{\mathrm{rev}}] = 450\ \mathrm{nmol} \cdot \mathrm{L}^{-1}$ and probe concentration $[Q] = 150\ \mathrm{nmol} \cdot \mathrm{L}^{-1}$.

Supplementary Figures

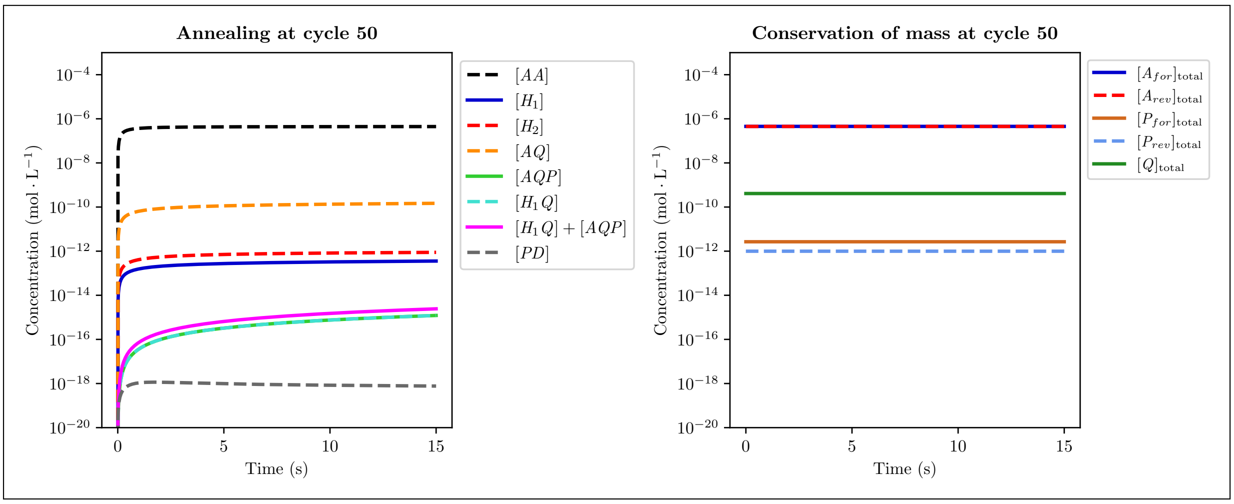

Supplementary Figure S1: **Concentrations of key molecular species and conservation of mass over time during the annealing step of PCR.**
In this simulation, we used the following parameters: number of cycles $N_{\text{cycles}} = 60$ and annealing temperature of 60°C; input double-stranded DNA concentration $[AA] = 10^7$ copies$\cdot\mu$L^{-1}; primer concentrations $[P_{\text{for}}] = [P_{\text{rev}}] = 450$ nmol $\cdot$ L^{-1}; probe concentration $[Q] = 150$ nmol $\cdot$ L^{-1}; hybridization rate constant $k_{\text{on}} = 10^6$ M^{-1}s^{-1} for all oligos (P_{for}, P_{rev}, Q); reannealing rate constant $k_{\text{r}}^{\text{on}} = 10^7$ M^{-1}s^{-1}; $k_{\text{r}}^{\text{off}} = 0$; $k_{\text{deg}} = 0, \beta = 1$, i.e., no thermal degradation of the Taq polymerase across cycles.

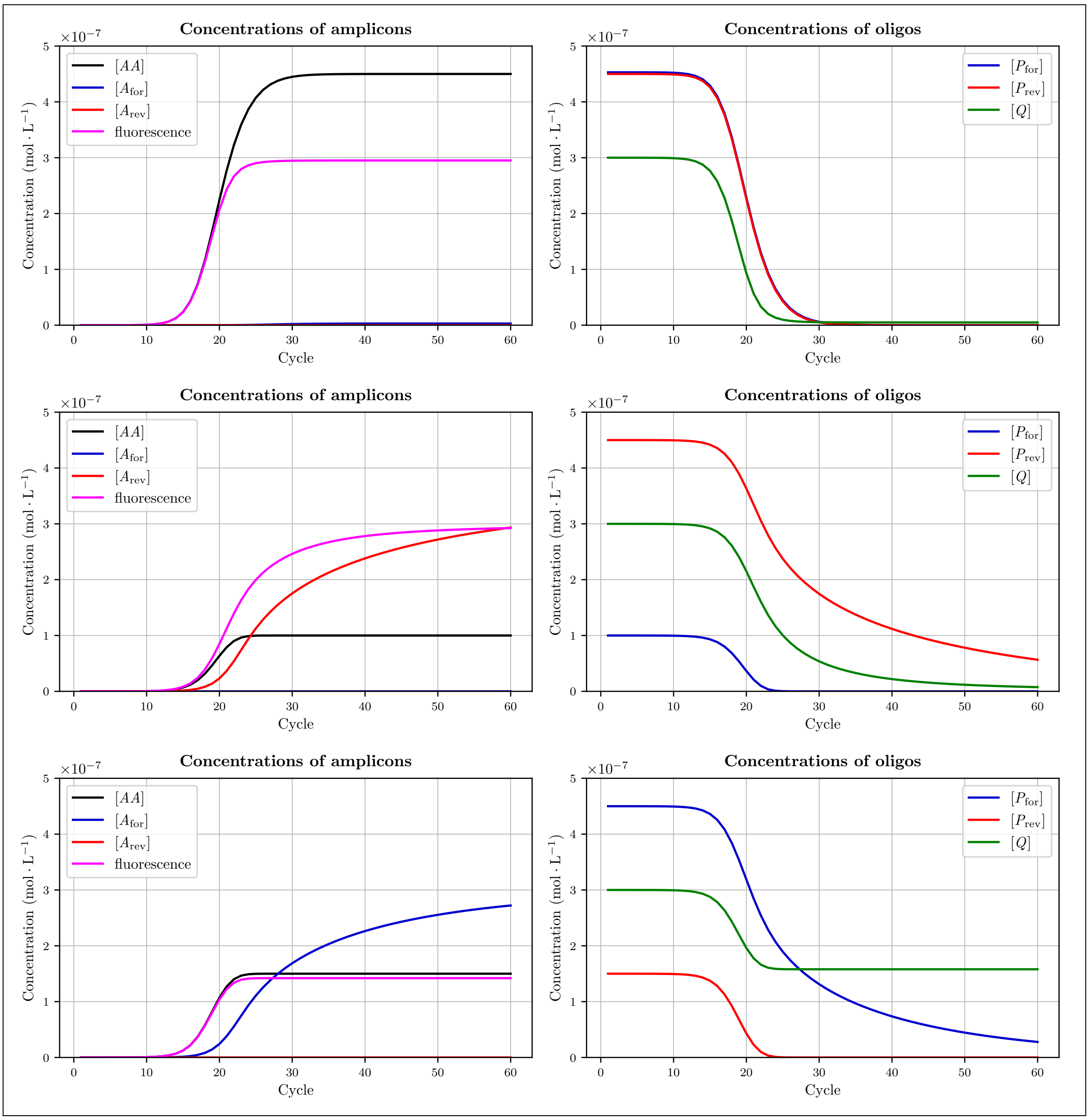

Supplementary Figure S2: **Simulated ACs, concentrations of AA, A_{for}, A_{rev}, P_{for}, P_{rev} and Q across PCR cycles obtained with atypical (including asymmetric) PCR designs.**

In these simulations, we ran real-time TaqMan PCRs for the C22 template, with the following parameters: $N_{\text{cycles}} = 60$ and $temp\_annealing = 60°\text{C}$, $[AA] = 10^7$ copies$\cdot\mu\text{L}^{-1}$, $k_{\text{on}} = 10^6\ \text{M}^{-1}\text{s}^{-1}$ for all oligos, $k_{\text{r}}^{\text{on}} = 10^7\ \text{M}^{-1}\text{s}^{-1}$; $k_{\text{r}}^{\text{off}} = 0$; $k_{\text{deg}} = 0, \beta = 1$, i.e., no thermal degradation of the Taq polymerase across cycles. We used the following oligo concentrations to exemplify how DYNAMIC can predict variations in AC features obtained in atypical PCR designs:

A — $[P_{\text{for}}] = [P_{\text{rev}}] = 450\ \text{nmol}\,\text{L}^{-1}$, $[Q] = 300\ \text{nmol}\,\text{L}^{-1}$;

B — $[P_{\text{for}}] = 100\ \text{nmol}\,\text{L}^{-1}$, $[P_{\text{rev}}] = 450\ \text{nmol}\,\text{L}^{-1}$, $[Q] = 300\ \text{nmol}\,\text{L}^{-1}$ (asymmetric PCR);

C — $[P_{\text{for}}] = 450\ \text{nmol}\,\text{L}^{-1}$, $[P_{\text{rev}}] = 150\ \text{nmol}\,\text{L}^{-1}$, $[Q] = 300\ \text{nmol}\,\text{L}^{-1}$ (asymmetric PCR).

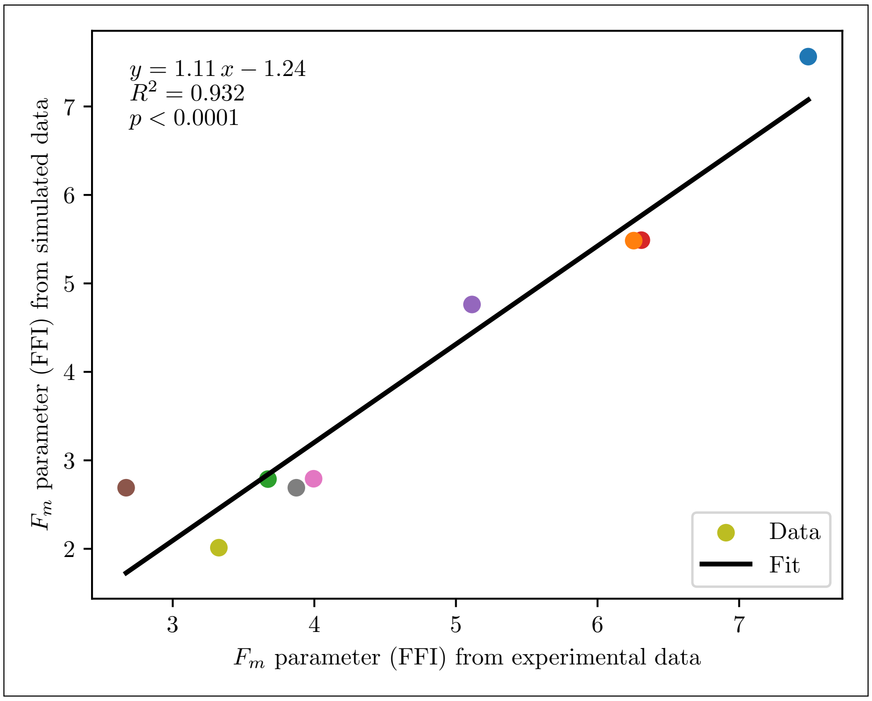

Supplementary Figure S3: **Correlation between FFI values of experimental and simulated data across nine primer mixes with varying concentrations of primers and probe.**

ACs obtained with nine primer mixes of varying concentrations of P_{for}, P_{rev} and Q were fitted to a 5-parameter sigmoid function, and F_m parameter values (i.e., the FFI) obtained with experimental and simulated data were compared using linear regression.

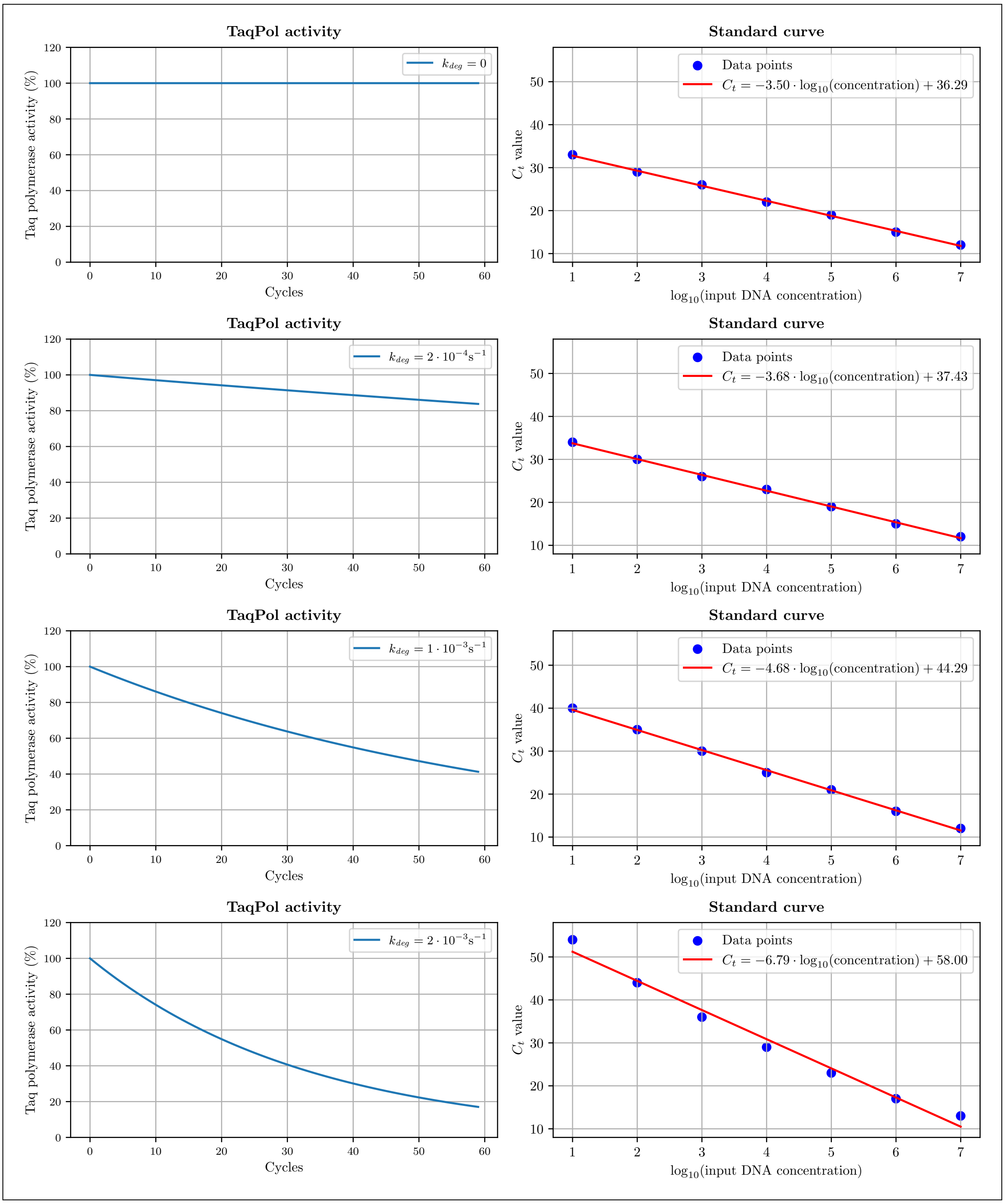

Supplementary Figure S4: **Relationship between Taq polymerase activity across cycles (thermal degradation) and standard curves (as predicted by DYNAMIC).**
From top to bottom, increasing k_{deg} values are associated with increasing thermal degradation of the Taq polymerase across PCR cycles, which impacts overall PCR efficiency (as assessed by computing the slope of standard curves).

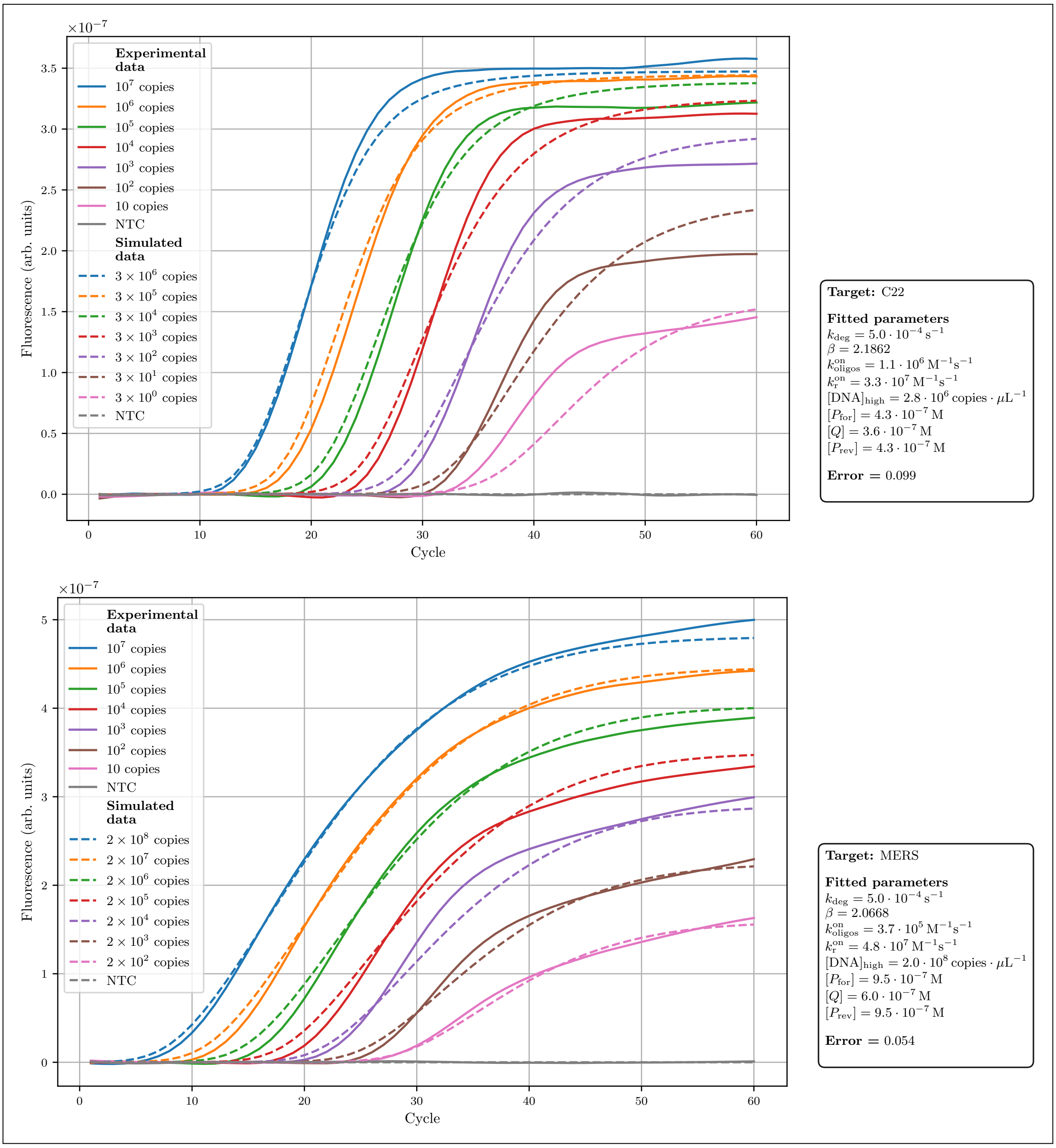

Supplementary Figure S5: **Simulated and experimental ACs across a DNA dilution series for the C22 and MERS templates.**
Experimental ACs (solid lines) are shown alongside simulated curves (dashed lines). Taq efficiency profile over cycles was modeled through a 2-parameter decay process. Averaged scaled MSE over 2×5 experiments 0.101 ± 0.002 and 0.056 ± 0.003, respectively.

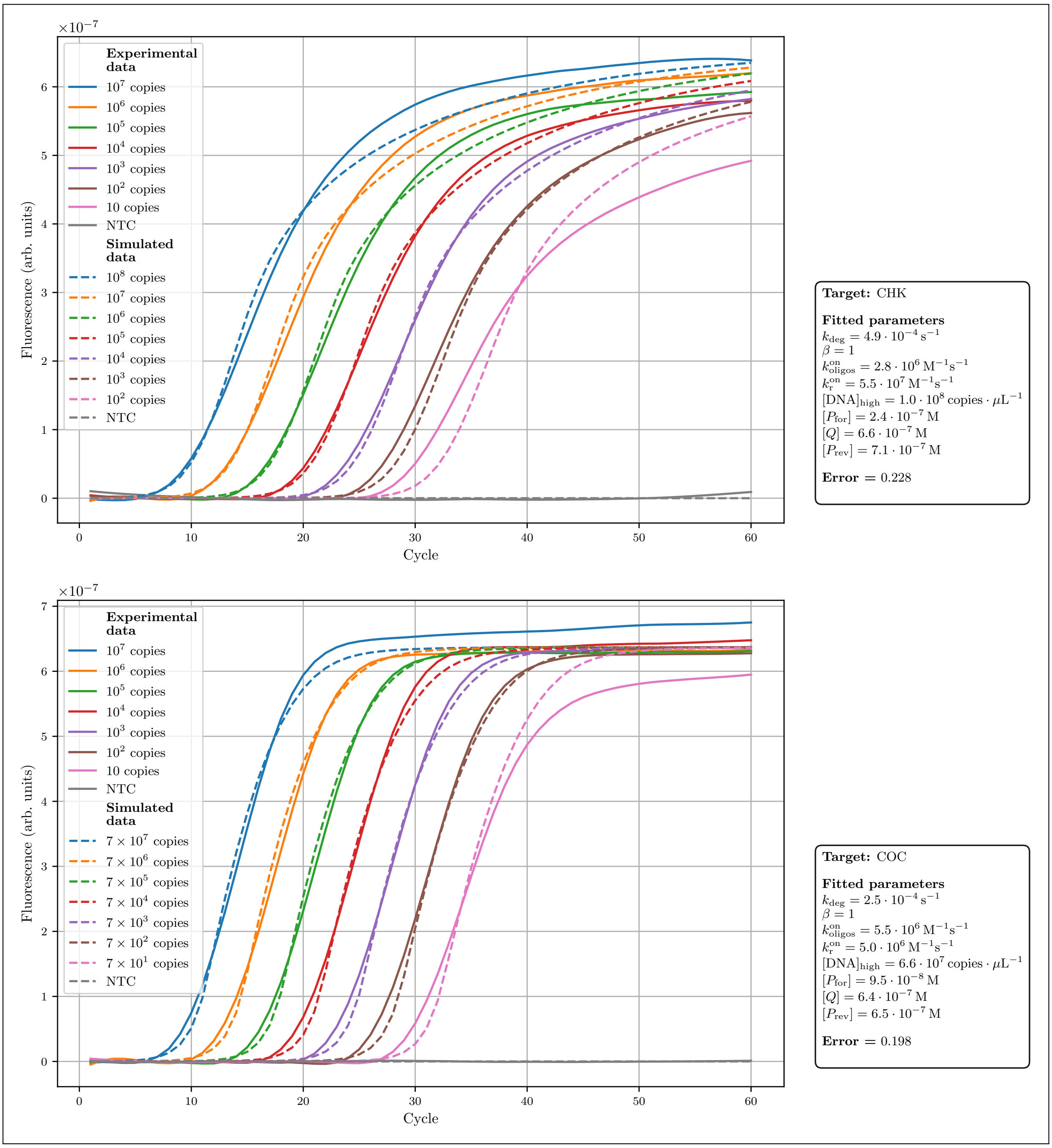

Supplementary Figure S6: **Simulated and experimental ACs across a DNA dilution series for the CHK and COC templates (Taq activity modeled through first-order decay process).**

Experimental ACs (solid lines) are shown alongside simulated curves (dashed lines). Taq loss of activity over cycles was modeled through a first-order decay process (β set to 1 for all experiments), resulting in decreased adequacy between predicted and experimental ACs: averaged scaled MSE over 2×5 experiments 0.228±0.00 and 0.198±0.00, respectively ($p = 0.003$ and $p = 0.007$ by Wilcoxon signed–rank test, in comparison to 2-parameter decay process with same targets; see Figure 8).

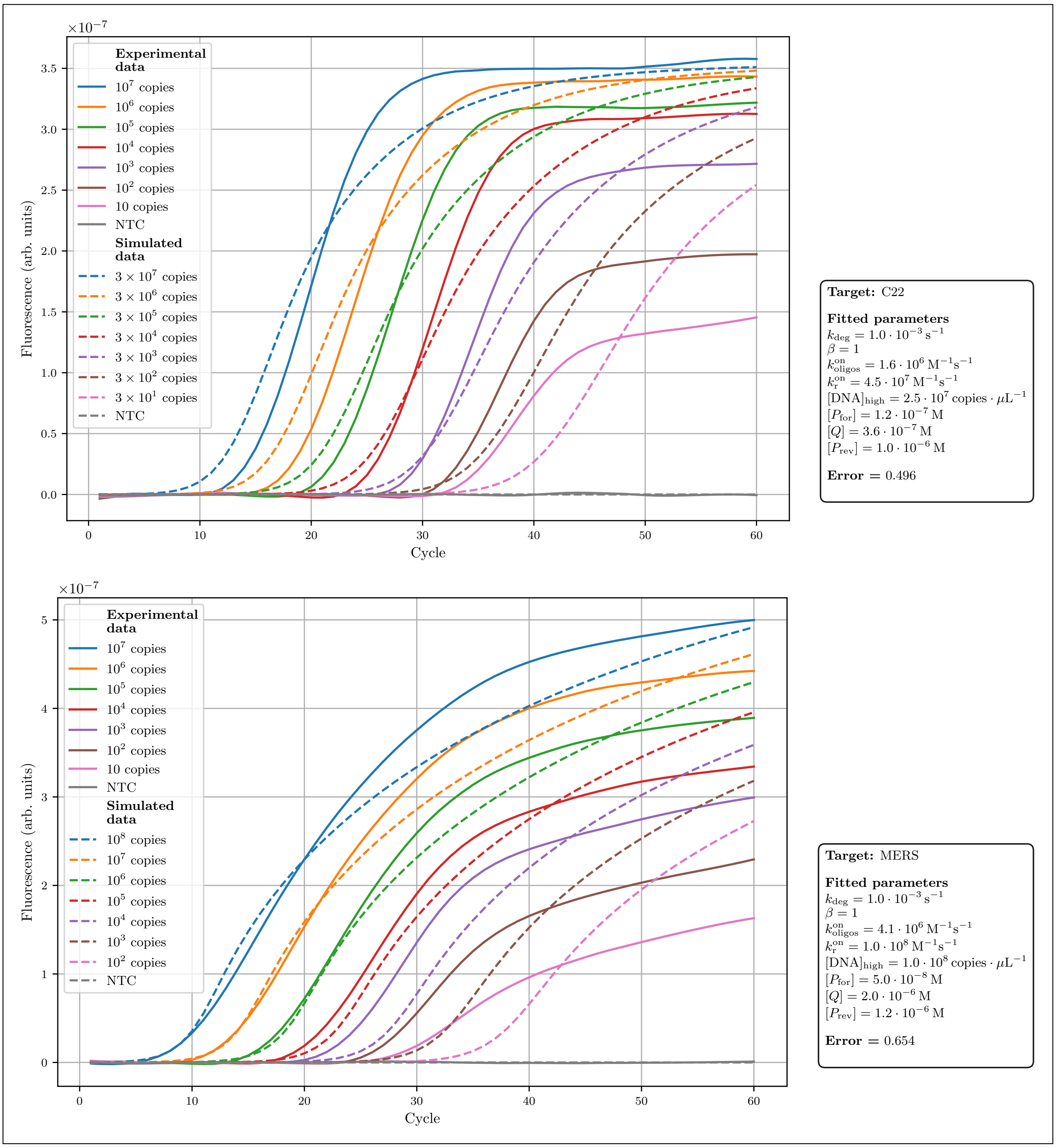

Supplementary Figure S7: **Simulated and experimental ACs across a DNA dilution series for the C22 and MERS templates (Taq activity modeled through first-order decay process).**

Experimental ACs (solid lines) are shown alongside simulated curves (dashed lines). Taq loss of activity over cycles was modeled through a first-order decay process (β set to 1 for all experiments), resulting in decreased adequacy between predicted and experimental ACs: averaged scaled MSE over 2×5 experiments 0.496±0.00 and 0.654±0.00, respectively ($p = 0.007$ and $p = 0.007$ by Wilcoxon signed–rank test, in comparison to 2-parameter decay process with same targets; see Supplementary Figure S5).
